# Adaptive Task-Oriented Locomotion Control of a 2D Planar Robotic Fish Model Using Deep Reinforcement Learning and Sensory-Feedback CPG Network

**DOI:** 10.3390/biomimetics11080534

**Published:** 2026-08-01

**Authors:** Gonca Ozmen Koca, Deniz Korkmaz, Cafer Bal, Mustafa Ay, Zuhtu Hakan Akpolat

**Affiliations:** 1 Department of Mechatronics Engineering, Faculty of Technology, Firat University, 23200 Elazig, Turkey; cbal@firat.edu.tr (C.B.); mustafaay@firat.edu.tr (M.A.); 2 Department of Electrical and Electronics Engineering, Faculty of Engineering and Natural Sciences, Malatya Turgut Ozal University, 44900 Malatya, Turkey; deniz.korkmaz@ozal.edu.tr; 3 Department of Electrical and Electronics Engineering, Faculty of Engineering, Fatih Sultan Mehmet Vakif University, 34015 Istanbul, Turkey; zhakpolat@fsm.edu.tr

**Keywords:** robotic fish, reinforcement learning, CPG, locomotion control, dynamic modeling, adaptive control

## Abstract

Autonomous locomotion in robotic fish requires task-dependent control capabilities under changing environmental conditions. This paper proposes a hierarchical simulation-based control framework for a two-joint robotic fish in a two-dimensional (2D) planar environment. This framework integrates the twin delayed deep deterministic policy gradient (TD3) algorithm with a sensory-feedback central pattern generator (CPG). A nonlinear planar dynamic model is designed as the learning environment, and a CPG network generates rhythmic undulatory swimming. The CPG network generates smooth locomotor patterns, while the TD3 policy performs high-level neuromotor modulation for task-dependent behavior. In the target-reaching benchmark, TD3–CPG achieves a 100.0% success rate with a Wilson 95% confidence interval (CI) of [96.30%, 100.00%], outperforming benchmark models. The proposed controller is also evaluated with obstacle avoidance in target reaching and station keeping under current disturbances. In circular obstacle avoidance, TD3–CPG achieves a 98.0% success rate and a 98.0% safe-pass rate, whereas the multiple rectangular obstacle scenarios yield an overall success rate of 91.7% over 96 trials. In station keeping, the controller achieves stay ratios of 87.57 ± 12.81% under constant current and 96.88 ± 10.79% under gust current, while keeping the mean target distance below the 0.25 m station keeping radius in both cases. Within the adopted 2D planar simulation environment, the obtained results demonstrate that the proposed method exhibits task-dependent maneuvering performance within the evaluated scenarios.

## 1. Introduction

Recently, central pattern generators (CPGs) have become a significant research area in biomimetic robots, enabling the generation of rhythmic swimming motions inspired by biological systems. In robotic fish control, CPG structure provides a biological framework for producing forward swimming, turning, orientation regulation, and motion responses to environmental stimuli [1,2,3]. In particular, closed loop CPG architectures supported by sensory-feedback have improved the adaptability of swimming behavior by enabling the modulation of rhythmic patterns according to environmental and task-related conditions. However, despite these advances, current CPG-based robotic fish systems are still limited in terms of speed, energy efficiency, maneuverability, and flexibility [4]. Although CPG is an effective mechanism for producing stable and periodic swimming patterns, it is not sufficient by itself for task-oriented and adaptive control in complex environments. Therefore, CPG-based control continues to serve as a robust motion generation baseline in robotic fish research, while also providing a suitable foundation for integration with sensory-feedback, decision-making mechanisms, and learning-based control strategies.

The development of CPG-based robotic fish control has gradually evolved from open loop rhythmic motion generation toward more integrated and task-aware control architectures. Biologically inspired oscillatory signals can generate stable swimming motions, and parameter optimization methods can improve the consistency in robotic fish prototypes [5]. Recent studies have extended this framework by incorporating sensory neurons, fuzzy logic, finite state machines, hierarchical decision structures, and center-of-gravity regulation to support autonomous swimming, obstacle avoidance, behavioral switching, and three-dimensional (3D) swimming modes [6,7,8,9]. These developments show that CPG can be used as a modular motion generation to provide high-level control. Nevertheless, recent reviews also emphasize that achieving biological levels of speed, efficiency, and maneuverability remains an open challenge, particularly when robotic fish must operate under uncertain, dynamic, or task-dependent environmental conditions [4]. However, CPG models still need more flexible and adaptive parameter modulation according to changing task requirements [5,10].

Reinforcement learning (RL) is a promising strategy for providing CPG-based control, ranging from rhythmic motion generation to adaptive and goal-oriented behavior. Unlike traditional control approaches, RL enables an agent to learn control policies through interaction with the environment, optimizing the task performance [11,12]. This capability is particularly important for the continuous adjustment of swimming behavior according to the task and energy-related constraints. The integration of deep reinforcement learning (DRL) with CPG allows for high-level adaptive modulation of control variables such as oscillation frequency, amplitude, and phase relationships, while preserving the rhythmic structure of biological swimming. Therefore, the CPG network produces stable and smooth locomotor patterns, and the RL policy can effectively determine the required swimming behavior. This combination provides a biologically inspired, learning-capable control framework for autonomous robotic fish navigation and control, integrating low-level swimming regularity with high-level decision adaptability [13,14,15]. RL approaches have attracted increasing attention in recent years for the control problem in underwater robots [16]. The main reason is that classical model-based methods often struggle to maintain the same level of performance under nonlinear current effects, environmental uncertainties, and real-time decision constraints. In this context, Zhan et al. [17] proposed a deep reinforcement learning policy to generate heading commands for a sonar target tracking an autonomous underwater vehicle (AUV) and showed that the method improved tracking performance. Compared with a heuristic strategy receding horizon control (RHC) approach, the proposed method achieved a 14.5% improvement in average tracking accuracy over the RHC approach, while reducing decision time from 9.8 s to under 0.1 s. However, this study mainly focuses on the general underwater decision-making problem and does not directly address the generation of biomimetic rhythmic swimming motion or the adjustment of CPG parameters for different task purposes. Therefore, while reinforcement learning appears to provide an effective decision mechanism for underwater vehicles, its integration with biomimetic motion control in robotic fish remains an important research issue. It is seen from the literature that reinforcement learning was initially used more for specific problems. Duraisamy et al. [18] investigated the speed tracking problem in robotic fish using a deep reinforcement learning model, compared the deep deterministic policy gradient (DDPG) and the twin delayed deep deterministic policy gradient (TD3) controllers, and showed that the TD3 structure exhibited more successful learning behavior by overcoming the local optimal problem. Similarly, Xu et al. [19] handled the online adjustment of body rigidity in a two-jointed robotic fish with a DDPG control approach and noted that this resulted in an improvement in thrust efficiency of approximately 9.77%. Both studies focus more on a single performance metric and do not discuss task-oriented behavior transitions together.

Feng et al. [20] studied cooperative tracing and capture by proposing a multi-agent reinforcement learning framework using a central training distributed execution structure for multiple robotic fish and demonstrated the applicability of this approach with both simulation and physical experiments. This study shows that reinforcement learning can be used in multi-agent and task-oriented underwater scenarios as well as single control problems. However, the focus of the study is on cooperative strategy learning among multiple robots. Considering studies in which reinforcement learning and CPG are used together, Chen et al. [11] proposed a control structure that can be trained in a real physical environment for the heading control of robotic fish. In this architecture, reinforcement learning is used at the decision layer, while CPG is responsible for generating periodic motion. In this way, decision-making and biomimetic rhythm generation are combined within the same framework. Experimental results showed that the proposed method achieved higher control accuracy and better stability than the classical proportional integral derivative (PID) control approach in complex current environments. However, the task considered in that study is mainly limited to heading direction control. Similarly, Su et al. [8] developed a two-layered structure combining CPG and DDPG for the control of the robot manta. In this structure, the CPG network generates rhythmic swimming motion at a low level, while in the high-level DDPG algorithm, the CPG control parameters are fit according to the state of the robot manta and velocity information. The study described how the proposed structure achieved tasks in the simulation environment and also reduced the task completion time compared to the CPG–PID approach in experiments. In this respect, the study can be considered as one of the current examples where reinforcement learning is directly related to CPG parameter adjustment. However, the task structure noted is mostly based on reaching the target point and swimming control. Wang et al. [21] suggested a predictive DDPG target tracking model for a biomimetic autonomous system and examined fixed targets, dynamic targets, and leader–follower tracking scenarios in a simulation environment. Likewise, Tian et al. [22] presented an improved deep Q network (DQN) path planning method that considers dynamic currents, terrain, and obstacles together and showed that path planning for bionic robotic fish can succeed with deep reinforcement learning. Although these studies demonstrate that reinforcement learning can be used effectively in target-oriented robotic fish tasks, their main focus is placed on high-level tracking or planning performance. The rhythmic generation of motion through CPG and the adaptation of such a structure for different tasks are not the primary focus of these studies.

When the CPG and DRL-based learning studies are analyzed, two open topics can be observed in the literature. First, CPG still plays a central role in generating biomimetic and continuous swimming movements. Secondly, DRL can be used to support decision-making, parameter adaptation, and control under ambiguous environmental conditions. Despite this progress, current studies focus either on a single task or on a limited control problem such as speed regulation, efficiency improvement, heading control, path following, or target tracking. A similar situation arises in studies combining RL with CPG, where parameter adjustment is usually addressed within the scope of a specific task. However, tasks such as target reaching by avoiding obstacles and maintaining a fixed position, although based on the same biomimetic swimming structure, need different control requirements in terms of environmental interaction, control objectives, and performance evaluation. This indicates that the adjustment of CPG parameters through RL structure according to task requirements has not yet been sufficiently extensively studied. Therefore, this study investigates how tuning CPG parameters through the RL mechanism affects robotic fish swimming performance under different task conditions.

To address the aforementioned issues, this study focuses on task-dependent adaptive locomotion control for a biomimetic robotic fish in a two-dimensional (2D) simulation environment through the integration of DRL and a sensory-feedback CPG architecture. The rhythmic swimming generation and task-oriented behavioral adaptation are designed with a common control framework. In the proposed methodology, a dynamic model provides the environment for learning, and a lamprey-inspired connectionist CPG network generates stable and rhythmic swimming motion. The sensory-feedback provides environment-dependent stimuli into the neural oscillators, and a TD3-based decision layer acts as a high-level neuromotor modulator that regulates task-relevant control variables according to the observed state. These components are organized hierarchically. The robotic fish dynamics determine the motion consequences of actuation, and the CPG network preserves continuous and biologically plausible propulsion. The sensory-feedback mechanism adapts the oscillatory response to local conditions, and the TD3 agent shapes the overall behavior for target reaching, obstacle avoidance, and station keeping. Therefore, the multitask learning problem involves the same locomotor backbone that is adaptively used under different task requirements. As a result, this study aims to formulate TD3 as a neuromotor modulation policy for a sensory-feedback CPG network and evaluate this architecture under different robotic fish behaviors using the same locomotor backbone. In this structure, the DRL policy does not directly control joint angles or torques, and it modulates CPG-related control variables. The main contributions of this paper can be summarized as follows:

- A hierarchical TD3–CPG locomotion framework is developed for biomimetic robotic fish control. The proposed architecture combines a nonlinear planar fish model, a lamprey CPG network with sensory-feedback, and a TD3-based high-level control layer, integrating biologically appropriate rhythmic actuation with learning-based task adaptation in a single control structure.
- CPG modulation is designed as a task-oriented learning problem. Instead of using CPG only as a fixed periodic motion generator, the network modulation is designed as a decision-making process, and the RL policy determines the oscillation variables based on task and environmental factors.
- A unified control framework is evaluated on three representative task behaviors. The adaptability of the proposed controller under challenging task conditions is evaluated through tasks such as reaching a target, reaching a target while avoiding obstacles, and station keeping under external current disturbances.
- A comprehensive evaluation is performed to examine convergence, safety, and disturbance tolerance under the neuromotor architecture. The obtained results show that TD3-CPG modulation enhances rhythmic swimming control with task-dependent adaptability.

The remainder of this paper is organized as follows: Section 2 presents details of background theories. The simulation results and performance comparisons are given in Section 3. Finally, Section 4 summarizes the conclusion of the study.

## 2. Materials and Methods

This section presents a hierarchical control methodology that bridges mathematical modeling with neuromorphic actuation and learning-based artificial intelligence. First, a comprehensive planar kinematic and nonlinear dynamic model is given to present the rigid body equations and quasi-steady hydrodynamic propulsive forces of the two-joint robotic fish. Later, a lamprey-inspired CPG model is presented with a sensory-feedback mechanism, which ensures instantaneous, reflex-like kinematic adaptation to exteroceptive environmental stimuli. The DRL approach given is utilized as a virtual brain system, and autonomously maps high-dimensional environmental state spaces to optimal descending neural commands, dynamically modulating the CPG network. Finally, the overall control framework is presented in detail.

### 2.1. Planar Kinematics and Nonlinear Dynamic Model of Robotic Fish

The motion of a two-joint robotic fish is modeled in a 2D plane to establish a computationally efficient and control-oriented environment for the DRL approach. The obtained dynamic model is derived from our previous robotic fish model based on real carp locomotion characteristics [23]. In the earlier study, the robotic fish model was represented as an articulated multibody system incorporating 3D kinematics and hydrodynamic interactions. In the present study, the body–tail propulsion and planar maneuvering effects are derived from the earlier study. For the DRL-based control, this model is reduced to a planar form while retaining the body–tail propulsion mechanism. The derived model representing the state transition dynamics of the Markov decision process (MDP), which serves as the interactive environment for the DRL agent, is reduced to a 3 degrees of freedom (3-DoF) planar system. The structure of the robotic fish consists of a rigid body of length $L_{b}$, a two-joint tail of lengths $L_{1}$ and $L_{2}$, and a passive caudal fin of length $L_{c}$. The lamprey CPG network generates rhythmic oscillatory motions by producing the angles of these joints. Two reference frames are utilized for the state-space variables of the DRL agent. The Earth fixed inertial frame $\left(O-X_{I}Y_{I}Z_{I}\right)\;$ defines the global position $\eta ={\left[\begin{array}{ccc} x & y & \psi \end{array}\right]}^{T}$ with the surge position, sway position, and yaw angle, respectively. The body-fixed frame $\left(0-x_{b}y_{b}z_{b}\right)$ placed at the center of mass (CoM) gives the local velocities, and it can be represented as $\nu ={\left[\begin{array}{ccc} u & v & r \end{array}\right]}^{T}$, including surge, sway, and yaw speeds. Therefore, the kinematic transformation can be given with the 3 × 3 Jacobian matrix [24]:

(1) $$\dot{\eta }=J\left(\psi \right)\nu$$

where $\dot{\eta }$ is the time derivative of the position vector in the inertial frame. Therefore, the kinematic transformation is expressed as follows:

(2) $$\left[\begin{array}{c} \dot{x}\\ \dot{y}\\ \dot{\psi } \end{array}\right]=\left[\begin{array}{ccc} \cos\left(\psi \right) & -\sin\left(\psi \right) & 0\\ \sin\left(\psi \right) & \cos\left(\psi \right) & 0\\ 0 & 0 & 1 \end{array}\right]\left[\begin{array}{c} u\\ v\\ r \end{array}\right]$$

The internal variables are defined by the link angle vector forming a serial tail structure as $\Theta ={\left[\begin{array}{cc} \theta_{1} & \theta_{2} \end{array}\right]}^{T}\in R^{2}$. The orientations of the tail links are $\emptyset ={\left[\begin{array}{cc} \emptyset_{1} & \emptyset_{2} \end{array}\right]}^{T}$ with $\emptyset_{1}=\psi +\theta_{1}$ and $\emptyset_{2}=\psi +\theta_{1}+\theta_{2}$. The link of positions $\left(r_{1},r_{2}\right)$ is used to obtain the hydrodynamic forces and can be given as:

(3) $$r_{1}=\left[\begin{array}{c} x\\ y \end{array}\right]+L_{1}\left[\begin{array}{c} \cos\left(\emptyset_{1}\right)\\ \sin\left(\emptyset_{1}\right) \end{array}\right]$$

(4) $$r_{2}=\left[\begin{array}{c} x\\ y \end{array}\right]+L_{1}\left[\begin{array}{c} \cos\left(\emptyset_{1}\right)\\ \sin\left(\emptyset_{1}\right) \end{array}\right]+L_{2}\left[\begin{array}{c} \cos\left(\emptyset_{2}\right)\\ \sin\left(\emptyset_{2}\right) \end{array}\right]$$

The rigid body kinetics and surrounding fluid body interactions are modeled using the Newton Euler formulation. The acceleration of the robotic fish displaces the surrounding water, creating an additional mass effect that is coupled with the rigid body mass. The 2D nonlinear dynamics of motion for the main body are defined as:

(5) $$M\dot{\nu }+C\left(\nu \right)\nu +D\left(\nu \right)\nu =\tau_{hyd}$$

Here, $M\in R^{3\times 3}$ is the generalized mass matrix including the rigid body mass matrix $M_{RB}$ and the hydrodynamic added mass matrix $M_{A}$. Also, $\tau_{hyd}={\left[\begin{array}{ccc} X & Y & N \end{array}\right]}^{T}$ is the generalized hydrodynamic force vector. Assuming the robotic fish design has an on-axis symmetry, and the CoM aligns with the origin of the body-fixed frame, the total mass matrix is diagonal. Therefore, the generalized mass matrix can be given as:

(6) $$M=\left[\begin{array}{ccc} m-X_{\dot{u}} & 0 & 0\\ 0 & m-Y_{\dot{v}} & 0\\ 0 & 0 & I_{z}-N_{\dot{r}} \end{array}\right]$$

where m is the total mass of the robotic fish, and $I_{z}$ is the rigid body of inertia. The added mass coefficients along the surge, sway, and yaw axes are $X_{\dot{u}}$, $Y_{\dot{v}}$, and $N_{\dot{r}}$, respectively. $C\left(\nu \right)\in R^{3\times 3}$ represents the Coriolis centripetal matrix, which captures the forces arising from the rotating body-fixed frame. This matrix integrates both rigid body $C_{RB}$ and added mass $C_{A}$. In addition, it is skew-symmetric to satisfy energy conservation properties and can be given as:

(7) $$C\left(\nu \right)=\left[\begin{array}{ccc} 0 & 0 & -\left(m-Y_{\dot{v}}\right)v\\ 0 & 0 & \left(m-X_{\dot{u}}\right)u\\ \left(m-Y_{\dot{v}}\right)v & -\left(m-X_{\dot{u}}\right)u & 0 \end{array}\right]$$

The hydrodynamic damping forces acting on the body, generated by friction and vortex effects, are defined by the damping matrix $D\left(\nu \right)\in R^{3\times 3}$. To represent speed-dependent resistance in a reduced-order form, $D\left(\nu \right)$ is decoupled into linear viscous and nonlinear quadratic damping components as $D\left(\nu \right)=D_{l}+D_{q}\left(\nu \right)$, and it can be obtained by:

(8) $$D\left(\nu \right)=\left[\begin{array}{ccc} X_{u}+X_{u\left|u\right|}\left|u\right| & 0 & 0\\ 0 & Y_{v}+Y_{v\left|v\right|}\left|v\right| & 0\\ 0 & 0 & N_{r}+N_{r\left|r\right|}\left|r\right| \end{array}\right]$$

where $X_{u}$, $Y_{v}$, and $N_{r}$ represent the linear damping coefficients, whereas $X_{u\left|u\right|}$, $Y_{v\left|v\right|}$, and $N_{r\left|r\right|}$ denote the nonlinear quadratic damping coefficients for the surge, sway, and yaw, respectively. The oscillatory motion of the tail links generates the propulsion of the robotic fish. To calculate the propulsive forces, a quasi-steady hydrodynamic model is given. For each tail link i, the local fluid speed vector $V_{i}$ at its geometric center is calculated by the speed of the main body ν and the relative kinematic speed induced by the angular motion of the joint:

(9) $$V_{i}=v+J_{tail,i}\left(\theta \right)\dot{\theta }$$

where $J_{tail,i}$ is the kinematic Jacobian mapping. The speed is divided into tangential and normal components as $V_{i,T}=V_{i}\cdot t_{i}$ and $V_{i,N}=V_{i}\cdot n_{i}$. Therefore, the hydrodynamic forces acting on the i-*th* link are presented with the normal $F_{i,N}$ and tangential $F_{i,T}$ drag components relative to the longitudinal axis of the link:

(10) $$F_{i,N}=-\frac{1}{2}\sigma C_{N}A_{i}V_{i,N}\left|V_{i,N}\right|$$

(11) $$F_{i,T}=-\frac{1}{2}\sigma C_{T}A_{i}V_{i,T}\left|V_{i,T}\right|$$

where σ is the fluid density, $A_{i}$ is the wetted surface area of the link, $C_{N}$ and $C_{T}$ are the drag coefficients. The total force vector acting on the link is given:

(12) $$F_{i}=F_{i,N}\cdot n_{i}+F_{i,T}{\cdot t}_{i}$$

Therefore, these forces are mapped to the CoM to obtain the generalized propulsion forces as follows:

(13) $$\tau_{hyd}=\left[\begin{array}{c} \begin{array}{c} \sum_{i=1}^{2}\left(F_{i,x}\right)\\ \sum_{i=1}^{2}\left(F_{i,y}\right) \end{array}\\ \sum_{i=1}^{2}\left(x_{i}F_{i,y}-y_{i}F_{i,x}\right) \end{array}\right]$$

Here, $\left(x_{i},y_{i}\right)$ is the position of the related link. These force terms provide a representation of tail-driven propulsion in the planar model. The motion of the robotic fish is generated by the CPG model, which directly produces rhythmic link angles. The output of the j-th CPG is defined as $y_{j}\left(t\right)$, and then the link angles can be given with:

(14) $$\theta_{j}\left(t\right)=\delta y_{j}\left(t\right)$$

**Figure 1:**
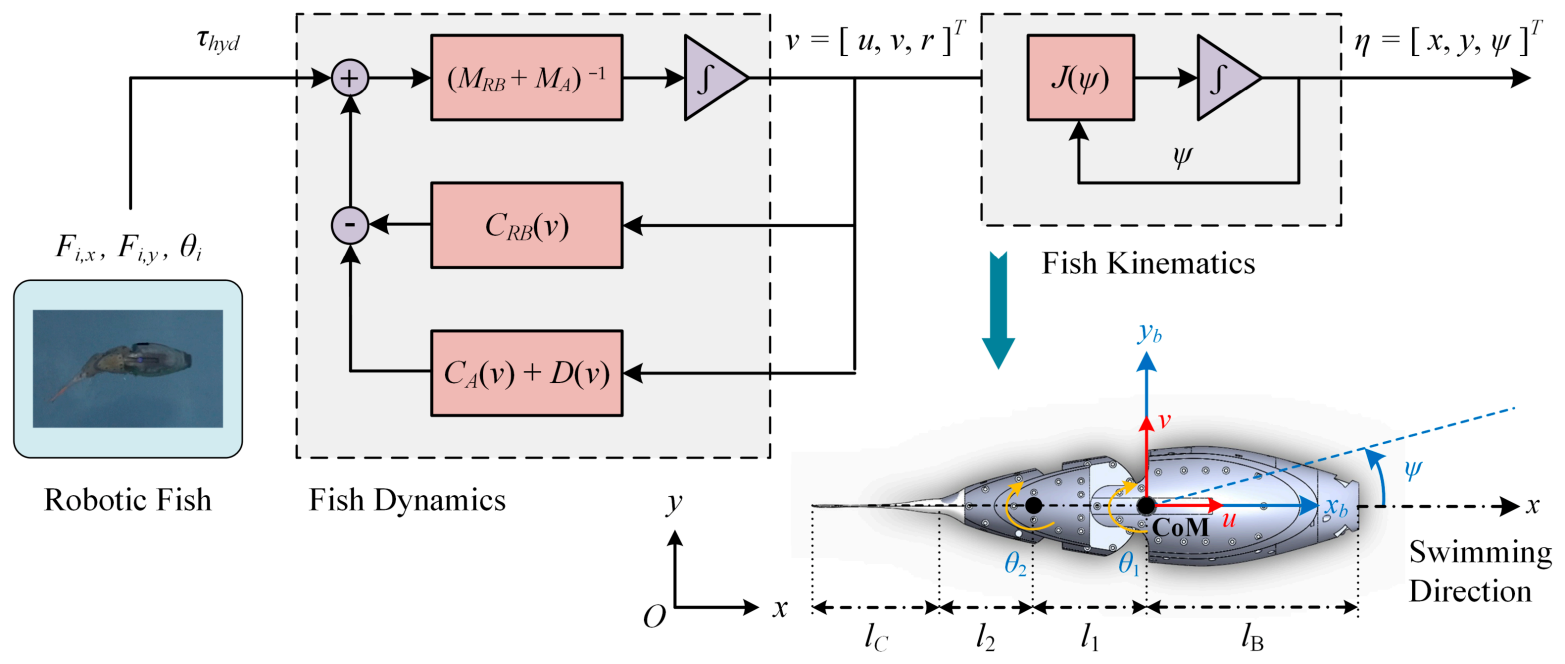
Robotic fish model.

Here, δ is the scaling coefficient. Therefore, the hydrodynamic forces are governed by the neural dynamics through the kinematic coupling between θ and ν. By modulating parameters in the CPG network via DRL, the agent handles nonlinear hydrodynamic forces and torques. Figure 1 shows the designed robotic fish model.

### 2.2. Lamprey CPG Model

Biological CPGs in the spinal cord behave as autonomous neural oscillators that generate rhythmic muscle activation patterns essential for complex locomotor behaviors such as walking, crawling, and swimming without external stimuli. In the context of control engineering, these neural models can offer a highly robust, stable, and adaptable output for the locomotion control of robotic fish, facilitating smooth and continuous transitions between diverse rhythmic swimming gaits in response to dynamic environmental conditions [25,26]. While CPG models are generally categorized into biophysical, connectionist, and abstract hierarchical levels, the connectionist approach is particularly advantageous because it leverages biologically plausible neuron models to preserve realistic network dynamics without the computational complexity associated with detailed biophysical simulations. The realization of effective autonomous motion control requires a framework capable of parameter modulation for gait generation, continuous sensory-feedback integration, and dynamic environmental adaptation. Although abstract-level CPG models frequently incorporate feedback inputs, their output cannot mimic the filtered, smooth transition characteristics found in biological kinematics.

This study adopts a connectionist-level CPG model with a sensory-feedback mechanism, inspired by the lamprey, one of the earliest known swimming vertebrates [27]. It can be noted that the CPG model used in this study is adopted from the authors’ previous lamprey-inspired CPG studies [6,7]. This framework can behave as the artificial neural spinal cord of the robotic fish. The CPG network is operated by a motor control unit (MCU) acting with a series of interconnected chain oscillators that collectively synthesize the rhythmic oscillation trajectories for propulsion. Operating like a brainstem, the MCU determines global phase relationships by propagating descending motor commands to modulate the subsegmental oscillators along the spinal chain. Each neural oscillator exhibits bilateral symmetry, divided into distinct left and right hemispheric segments. Within each symmetrical part, the dynamic interactions of three specialized interneurons are called the crossed interneuron (CIN), lateral interneuron (LIN), and excitatory interneuron (EIN). Finally, a motor neuron (MN) controls the CPG output. Rhythmic oscillation activities occur through continuous interactions between these nerve units.

In the CPG network, oscillators are connected via external coupling synapses. The EINs send inhibitory synapses to neighboring oscillators, while each LIN is configured to receive these synaptic signals. It can be noted that this coupling topology preserves laterality. Presynaptic and postsynaptic connections between distinct oscillators also remain confined to their respective left or right domains. The calculation of these interneurons is derived from the leaky integrate and fire (LIF) model, which provides a computationally efficient and biological representation of temporal membrane potential variations. The rhythmic output of an oscillator generates the nonlinear spatial and temporal dynamics, and it is mathematically formulated through a system of coupled equations. The temporal membrane potentials for the CIN and LIN are described as follows:

(15) $$\tau {\dot{x}}_{i}^{\left(CIN\right)}=-x_{i}^{\left(CIN\right)}+\sum_{k=1}^{N}\omega_{i,k}s_{i,k}^{\left(CIN\right)}$$

(16) $$\tau {\dot{x}}_{i}^{\left(LIN\right)}=-x_{i}^{\left(LIN\right)}+\sum_{k=1}^{N}\omega_{i,k}s_{i,k}^{\left(LIN\right)}+\sum_{j=1}^{N}\omega_{j,\left(j-1\right)}c_{\left(j-1\right)}^{\left(LIN\right)}$$

where τ denotes the temporal period of the oscillator. The subscript i indicates the segment laterality, k represents the index of the presynaptic neuron within the same oscillator, and N is the total number of connected intersegmental neurons. The variables $x^{\left(CIN\right)}$ and $x^{\left(LIN\right)}$ define the membrane potentials of the CIN and LIN, respectively. The intersegmental synaptic weight from neuron k to neuron i is denoted by $\omega_{i,k}$, while $s_{i,k}$ represents the propagated presynaptic potential within the same segment. For the intersegmental coupling in the LIN dynamics, the index j represents the specific oscillator position along the spinal chain. Consequently, $\omega_{j,\left(j-1\right)}$ defines the synaptic weight from the previous oscillator, and $c^{\left(CIN\right)}$ is the corresponding neurotransmitter potential propagated from that neighboring oscillator.

The EIN incorporates a nonlinear sigmoid activation mechanism, and this allows for continuous variation in the membrane potential and strong propagation of excitatory signals. The sigmoid function is scaled with the oscillation amplitude A and symmetrically shifted, receiving constitutive input from the opposite CIN as:

(17) $$x_{i}^{\left(EIN\right)}=\frac{A}{1+e^{x^{\left(\bar{CIN}\right)}}}-\frac{A}{2}$$

where $x^{\left(EIN\right)}$ is the membrane potential of the EIN. In the simulations, A is defined as 20°. The $x^{\left(\bar{CIN}\right)}$ is the membrane potential of the contralateral CIN. Therefore, the motor neuron dynamics $x^{\left(MN\right)}$ can be expressed as follows:

(18) $$\tau {\dot{x}}_{i}^{\left(MN\right)}=-x_{i}^{\left(MN\right)}+\beta A+\sum_{k=1}^{N}\omega_{i,k}s_{i,k}^{\left(MN\right)}$$

**Figure 2:**
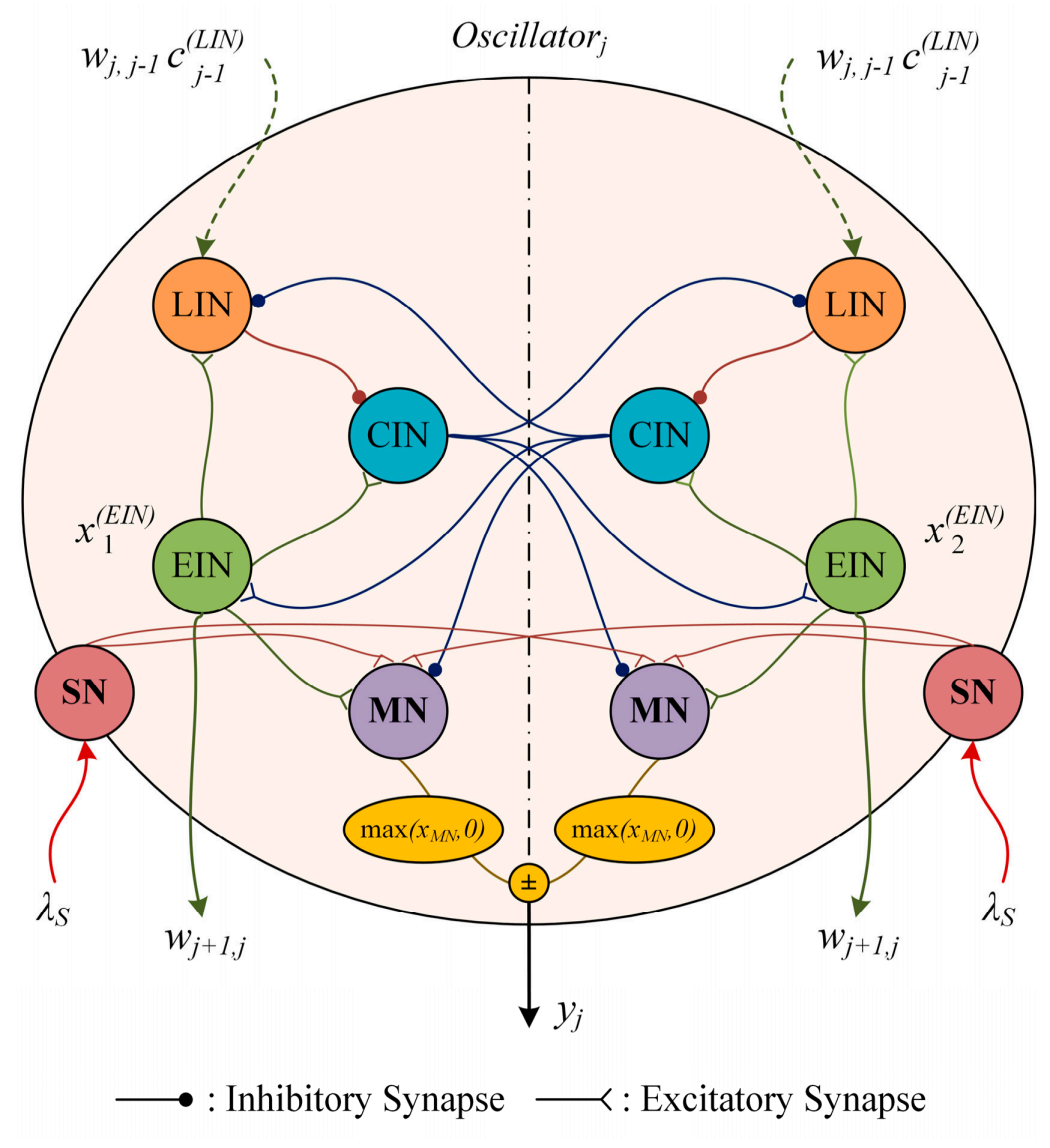
Schematic diagram of the CPG model. Each CPG oscillator is connected via unidirectional synaptic weights from the EIN to the LIN.

Here, β is the offset parameter. These computations culminate in the coordinated output of the j-th oscillator, expressed as:

(19) $$y_{j}=\max\left(x_{1}^{\left(MN\right)},0\right)-\max\left(x_{2}^{\left(MN\right)},0\right)$$

where $y_{j}$ serves as the oscillatory output signal. The steady state convergence of this structure guarantees continuous rhythmic outputs. To mimic the biological propulsion of Carangiform fish, a phase delay is constructed, characterized by a body wave that travels through the body and rises smoothly to the caudal fin. The MCU regulates this phase relationship by propagating targeted inhibitory synapses along the spinal cord. This modulation is achieved through the integration of two high-level synapses, $\omega_{L,R}$ and $\omega_{R,L}$, localized within the MCU. The inhibitory synapse $\omega_{R,L}\in \left(0\right.,\left.1\right]$ projects from the left EIN to the right LIN, while the excitatory synapse $\omega_{L,R}\in \left[-1\right.,\left.0\right)$ connects the right EIN to the left LIN. These weights are calculated using the phase correlation coefficients as follows:

(20) $$\left(\omega_{L,R}\right.,\;\left.\omega_{R,L}\right)=\left(\frac{\alpha }{\alpha +\gamma }\right.,\;\left.\frac{-\gamma }{\alpha +\gamma }\right)$$

where α and γ are the phase correlation coefficients bounded within $\left(0\right.,\left.1\right]$. To guarantee the smooth and stable sinusoidal oscillation, internal synaptic weights are normalized, while the projection from EIN to MN is scaled to 0.1. Furthermore, the system relies on asymmetric initialization. Figure 2 presents the schematic diagram of the CPG model.

### 2.3. Sensory-Feedback Mechanism

The robotic fish requires an adaptive detection layer capable of instantly integrating external and internal signals to navigate in aquatic environments with complex dynamic structures [28]. During locomotion, the obtained environmental data is converted into a normalized external stimulus input parameter, denoted as λ. This stimulus is directly transmitted to sensory neurons (SNs) embedded in the CPG model, allowing the neural network to dynamically adjust the rhythmic output transmitted to the tail link via MNs, and kinematic adaptation based on desired control behaviors can be achieved. The sensory-feedback mechanism filters environmental noise and responds only to critical changes with a threshold-based activation. Therefore, the nonlinear state dynamics of each sensory sensor are obtained using differential equations with time constants as follows:

(21) $$\begin{array}{c} \tau_{res}{\dot{x}}_{i}^{\left(SN\right)}\\ \tau_{rec}{\dot{x}}_{i}^{\left(SN\right)} \end{array}=\left\{\begin{array}{cc} -x_{i}^{\left(SN\right)}+\rho \lambda ,\;\; & \left(if\;\lambda \geq \Omega \right)\\ -x_{i}^{\left(SN\right)},\;\; & \left(otherwise\right) \end{array}\right.$$

where $x^{\left(SN\right)}$ characterizes the membrane potential of the sensory neuron. $\tau_{res}$ and $\tau_{rec}$ also define the response and recovery time constants of the stimulus. The variable ρ acts as the stimulus scaling coefficient, and λ determines the critical activation threshold Ω that can be exceeded to elicit a neural response. Consequently, when the stimulus amount reaches the threshold, the SN dynamically activates and propagates highly sensitive excitatory synaptic activity to all associated MNs. Without sufficient stimulation, the SN state degrades and produces zero output. In this study, the optimal parameter configuration for the sensory mechanism is determined as $\tau_{rec}=0.4$, $\tau_{res}=0.1095$, and ρ = 2, respectively. These sensory parameters are used in both the left and right segments of the CPG network to ensure symmetrical modulation.

### 2.4. Task-Oriented TD3 Structure for Neuromotor Modulation

Given the nonlinear dynamics of the robot fish, the RL framework is designed as a high-level cognitive decision-making layer governing the CPG network in the spinal cord. The TD3 algorithm [29] is applied to effectively navigate the robot and operate in the continuous action control domain. The TD3 agent continuously evaluates environmental feedback to generate descending neural commands adapted for target search, obstacle avoidance, and station keeping against the current disturbances. The CPG network provides stable and rhythmic outputs using these modulatory signals. Therefore, the hierarchical architecture integrates high-level neuromotor modulation with CPG-based locomotion, enabling efficient and task-oriented control. In this learning problem, each locomotion task is modeled as an MDP:

(22) $$\begin{array}{cc} M^{\left(j\right)}=\left(S^{\left(j\right)},\;{Ą}^{\left(j\right)},{Ƥ}^{\left(j\right)},r^{\left(j\right)},\gamma \right), & j\in \left\{1,2,3\right\} \end{array}$$

where j defines the related tasks of the robotic fish. The parameters S, Ą, Ƥ, r, and γ∈(0,1) are the task-dependent state space, action space, transition probability induced by the nonlinear fish dynamics and CPG modulation, immediate reward, and discount factor, respectively. For each task, a deterministic policy $\pi_{\emptyset }^{\left(j\right)}$ with parameters ∅ is optimized to maximize the expected discounted return as:

(23) $$J\left(\pi_{\emptyset }^{\left(j\right)}\right)=E\left[\sum_{k=0}^{K-1}\gamma^{k}r_{k}^{\left(j\right)}\right]$$

where k is the control step. The RL layer provides a set of instructions that direct and regulate the oscillation regime. For the first two tasks, the action is scalar, and sensory modulation sends a neural command that is converted to $\lambda_{k}$. The task set can be given as:

(24) $$a_{k}^{\left(1,2\right)}\in \left[-1,1\right]$$

(25) $$\begin{array}{cc} a_{k}^{\left(3\right)}\in \left[\begin{array}{c} a_{\lambda ,k}\\ a_{f,k} \end{array}\right], & a_{\lambda ,k},\;a_{f,k}\in \left[-1,\;1\right] \end{array}$$

Here, $a_{\lambda ,k}$ controls the turning bias and $a_{f,k}$ modulates the oscillation frequency of the CPG. Therefore, the RL policy remains low-dimensional, whereas the rhythmic locomotor pattern is generated by the CPG dynamics. For the target search and obstacle avoidance, the policy is mapped to the bias through a rate-limited update as:

(26) $$\lambda_{k}=sat\left(\lambda_{max}a_{k},\lambda_{k-1}-∆\lambda_{max},\lambda_{k-1}+∆\lambda_{max}\right)$$

Here, $\lambda_{max}$ is the task-specific maximum external stimulus. This formulation allows for smoother turning transitions by preventing sudden changes. The same structure is maintained in obstacle avoidance, and the turning authority is expanded to accommodate more aggressive maneuvers. For station keeping, the two actions are mapped as:

(27) $$\lambda_{k}=\forall \left(\lambda_{max}tanh\left(a_{\lambda ,k}\right),\lambda_{k-1},∆\lambda_{max}\right)$$

(28) $$\kappa_{k}=1+k_{f}tanh\left(a_{f,k}\right)$$

where $\kappa_{k}$ is the instantaneous frequency gain, and $k_{f}$ is the modulation coefficient. The frequency gain is then passed to the CPG for scaling of the intrinsic time constants as follows:

(29) $$\tau_{k}=\frac{\tau_{0}}{\kappa_{k}},\;\;\;\;\tau_{res,k}=\frac{\tau_{res,0}}{\kappa_{k}},\;\;\;\;\tau_{rec,k}=\frac{\tau_{rec,0}}{\kappa_{k}}$$

where $\tau_{0}$, $\tau_{res,0}$, and $\tau_{rec,0}$ are the nominal oscillator, response, and recovery time constants, respectively. Therefore, the agent can simultaneously regulate heading through $\lambda_{k}$ and propulsion intensity through $\kappa_{k}$, which is essential for maintaining position against a current. For the target search, the observation vector can be constructed as:

(30) $$s_{k}^{\left(1\right)}={\left[\begin{array}{cccccccccccc} u_{k} & v_{k} & r_{k} & \cos\left(\psi_{k}\right) & \sin\left(\psi_{k}\right) & \cos\left(e_{\psi ,k}\right) & \sin\left(e_{\psi ,k}\right) & d_{k} & {\hat{f}}_{k} & {\hat{A}}_{k} & {\dot{y}}_{tail,k} & \lambda_{k-1} \end{array}\right]}^{T}$$

Here, $e_{\psi ,k}$, $d_{k}$, ${\hat{f}}_{k}$, and ${\hat{A}}_{k}$ are the heading error, Euclidean distance to the target, predicted tail frequency, and oscillation amplitude, respectively. ${\dot{y}}_{tail,k}$ is the filtered lateral tail speed. By combining these observations, the policy ensures that navigation is aligned with the current oscillation regime. For target reaching with obstacle avoidance, any obstacle is detected with the sensor beam information, and the state vector can be given by:

(31) $$s_{k}^{\left(2\right)}={\left[\begin{array}{ccccccc} s_{k}^{\left(1\right)T} & b_{k}^{T} & d_{min,k} & {∆}_{LR,k} & \cos\left(e_{obs,k}\right) & \sin\left(e_{obs,k}\right) & {\bar{d}}_{min,k} \end{array}\right]}^{T}$$

Here, $b_{k}^{T}\epsilon {\left[0,\;1\right]}^{N_{b}}$ is the normalized sensor output, and $N_{b}$ is the sensor beam. $d_{min,k}$ is the minimum clearance to obstacle surfaces, and ${∆}_{LR,k}$ is the left and right beam asymmetry. $e_{obs,k},$ and ${\bar{d}}_{min,k}$ also represent the minimum distance to the obstacle surfaces and the normalized clearance with respect to the sensing range. This representation allows the policy to provide a balance between achieving the goal and the margin of safety at the same decision-making stage. In station keeping, the observation vector is the same as in Equation (30). The policy deduces the presence and effect of current from motion, distance error, body speeds, and the dynamics of the CPG network. Accordingly, the observation vector used for target reaching and station keeping has 12 dimensions. For obstacle avoidance, the 11 range-beam measurements and four obstacle-related geometric variables are added to the same base observation structure. Therefore, the obstacle avoidance observation vector has 27 dimensions in total. Reward functions are structured to reflect the different behavioral goals of tasks. For the target search, the reward function can be given as follows:

(32) $$r_{k}^{\left(1\right)}=w_{d}\left(d_{k-1}-d_{k}\right)+w_{h}\cos\left(e_{\psi ,k}\right)+w_{\nu }\nu_{P,k}-w_{s}\nu_{k}^{2}-w_{r}r_{k}^{2}-w_{\lambda }\lambda_{k}^{2}-w_{∆\lambda }{\left(∆\lambda_{k}\right)}^{2}-w_{e}{\dot{y}}_{tail,k}^{2}-w_{t}+R_{g}ℾ\left(d_{k}\leq r_{g}\right)$$

Here, while the positive w are the related reward weights, negatives are the penalty weights. $\nu_{P,k}$ is the world frame swimming speed and $R_{g}$ is the goal reward. $ℾ\left(\cdot \right)$ is also the indicator function. Furthermore, if there is no significant progress within the specified time frame, early termination is triggered, preventing the robot from swimming away from the target area. The obstacle avoidance reward extends Equation (32) by adding explicit safety and free-space terms as:

(33) $$r_{k}^{\left(2\right)}=r_{k}^{\left(1\right)}+w_{f}\cos\left(e_{free,k}\right)-w_{0}\max\left(\cos\left(e_{obs,k},0\right)\right)\rho_{k}-w_{n}\eta_{k}+w_{c}{∆d}_{min,k}^{+}+R_{cp}ℾ\left(C_{k}\right)+R_{coll}ℾ\left(d_{min,k}\leq d_{coll}\right)$$

where $e_{free,k}$, $\rho_{k}$, and $\eta_{k}$ are the heading error relative to a composite free-space direction, the proximity factor, and the normalized near-obstacle penalty, respectively. ${∆d}_{min,k}^{+,}$ and $d_{coll}$ are also rewards, clearance recovery, and the collision threshold. The composite free-space direction is calculated from the target vector and the repulsive field generated by environmental obstacles as follows:

(34) $$d_{free,k}=\frac{{\hat{g}}_{k}+\beta \sum_{i=1}^{N_{0}}\frac{p_{k}-c_{i}}{{\left(d_{i,k}^{clr}+\varepsilon \right)}^{2}\left\|p_{k}-c_{i}\right\|}}{\left\|{\hat{g}}_{k}+\beta \sum_{i=1}^{N_{0}}\frac{p_{k}-c_{i}}{{\left(d_{i,k}^{clr}+\varepsilon \right)}^{2}\left\|p_{k}-c_{i}\right\|}\right\|}$$

Here, ${\hat{g}}_{k}$, $p_{k}$, $c_{i}$, $d_{i,k}^{clr}$, and $N_{0}$ are the unit goal direction, fish position, center of the obstacle, clearance to the obstacle surface, and number of obstacles, respectively. This reward structure not only penalizes collision but also sustains target seeking behavior and rewards avoidance behavior. In the station keeping strategy, the reward prioritizes maintaining a fixed position around the starting point rather than completing the entire route. This reward can be expressed as:

(35) $$r_{k}^{\left(3\right)}=w_{app}^{SK}\left(d_{k-1}-d_{k}\right)-w_{dist}^{SK}d_{k}+w_{st}^{SK}ℾ\left(d_{k}\leq r_{g}\right)-w_{v}^{SK}v_{k}^{2}-w_{r}^{SK}r_{k}^{2}-w_{e}^{SK}{\dot{y}}_{tail,k}^{2}-w_{t}^{SK}$$

where $d_{k}$ represents the distance to the station keeping point. The variables $w_{app}^{SK}$, $w_{dist}^{SK}$, $w_{st}^{SK}$, $w_{v}^{SK}$, $w_{r}^{SK}$, $w_{e}^{SK}$, and $w_{t}^{SK}$ are the weights of the approach reward, distance penalty, stay region reward, speed penalty, yaw rate penalty, tail motion energy penalty, and time penalty, respectively. Unlike previous tasks, the terminal objective reward is not required. Instead, the agent is encouraged to remain within a small, unchanging area centered at the origin. The TD3 algorithm is adopted to solve these continuous action control problems. The policy network ${\bar{\pi }}_{\emptyset }$ and the two critic networks $Q_{\theta_{1}}$ and $Q_{\theta_{2}}$ are implemented as fully coupled multilayer perceptrons containing two hidden layers of 128 neurons and ReLU activation, along with a tanh output layer in the actor. The critics predict the action value with the Q-learning, while the target policy correction adjusts the bootstrapped target. The TD3 target is given by:

(36) $$y_{k}=r_{k}+\gamma \underset{i\in \left\{1,2\right\}}{\min}Q_{{\bar{Q}}_{i}}\left(s_{k+1},{\bar{\pi }}_{\bar{\emptyset }}\left(s_{k+1}\right)+\epsilon_{k}\right)$$

where ${\bar{\pi }}_{\bar{\emptyset }}$ and $Q_{{\bar{Q}}_{i}}$ are the target networks, and $\epsilon_{k}$ is the Gaussian smoothing noise. Then, the critic parameters can be updated by:

(37) $$L\left(\theta_{i}\right)=\frac{1}{N}\sum_{n=1}^{N}{\left(Q_{\theta_{i}}\left(s_{n},a_{n}\right)-y_{n}\right)}^{2}$$

The actor is optimized through the deterministic policy gradient as:

(38) $$\nabla_{\emptyset }J\left(\emptyset \right)\approx \frac{1}{N}\sum_{n=1}^{N}\nabla_{a}Q_{\theta_{1}}\left(s_{n},a\right){|}_{a=\pi_{\emptyset }\left(s_{n}\right)}\nabla_{\emptyset }\pi_{\emptyset }\left(s_{n}\right)$$

Since the control variables are continuous and low-dimensional, the combination of twin critics provides a stable optimization mechanism. Overall, the proposed RL approach provides task-dependent modulation on the neuromechanical structure. Therefore, the TD3 policy specifies how descending commands should be adapted online to ensure target reaching, safe obstacle avoidance, and station keeping. The controller maintains interpretability at the action level while preserving the adaptability of deep reinforcement learning. The details of the reward function coefficients used in the DRL training process are given in Table A1.

### 2.5. Framework of the Control Approach

The designed approach combines a sensory-feedback CPG network and a deep reinforcement-learning layer within a hierarchical, task-adaptive architecture. The main purpose of the structure is to enable autonomous adaptation of the robotic fish during target reaching, obstacle avoidance, and position maintenance. While the CPG network produces stable oscillatory motor patterns, the TD3 policy serves as a high-level neuromotor decision module that regulates oscillatory swimming based on the current task and environmental information. The closed loop control framework can be summarized as:

(39) $$x_{k+1}^{j}=F^{j}\left(x_{k}^{j},\pi_{\emptyset }^{j}\left(H^{j}\left(x_{k}^{j},{Ε}_{k}\right)\right),{Ε}_{k}\;\right)$$

**Figure 3:**
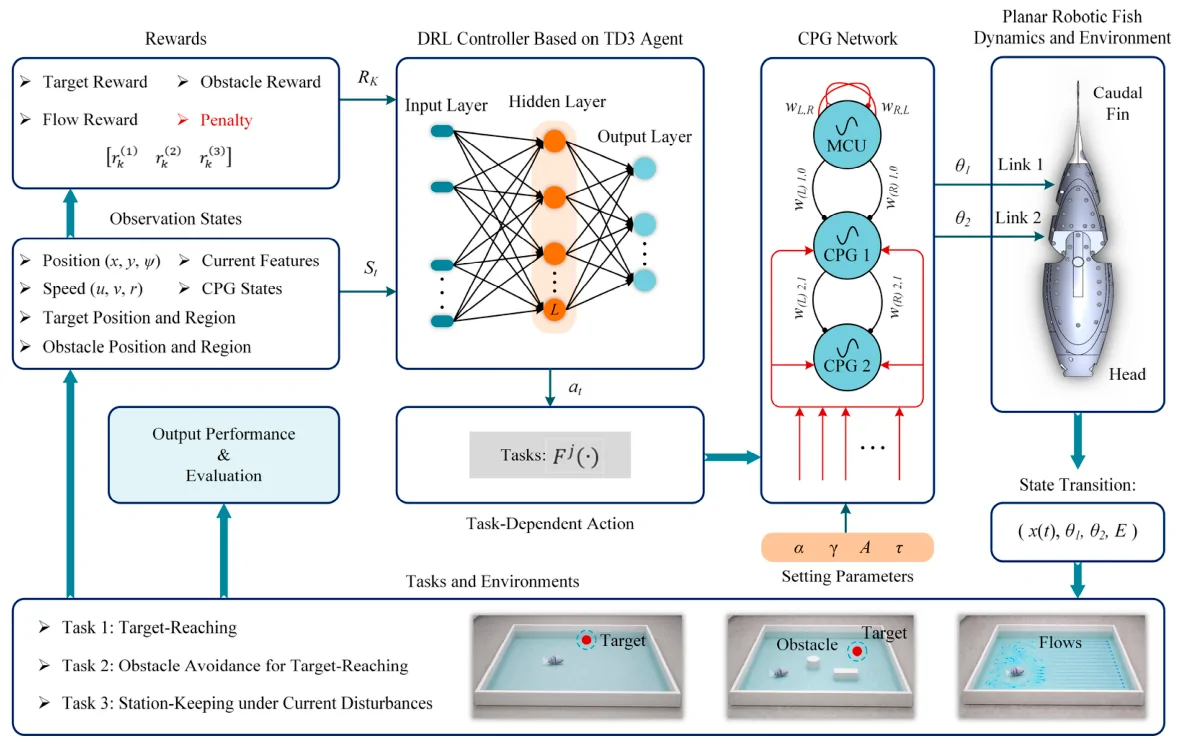
Overall framework of the control approach.

Here, $x_{k}^{j}$ is the task-dependent closed loop state, $H^{j}\left(\cdot \right)$ is the observation generation operator, $\pi_{\emptyset }^{j}\left(\cdot \right)$ is the task-specific TD3 policy, ${Ε}_{k}$ is the environmental state, and $F^{j}\left(\cdot \right)$ is the overall transition generated by the sensory modulation, CPG response, and fish dynamics. Figure 3 shows the overall framework of the proposed control architecture.

At each control step, the robotic fish extracts task-related information from the environment and motion patterns. These inputs include target orientation, body velocities, directional information, obstacle-related geometric information, and rhythmic variables derived from the CPG response. The observation is then processed by the TD3 agent. In the target reaching and obstacle avoidance tasks, the learned command regulates the β parameter for turning. In station keeping, the learned controller also adjusts the oscillation frequency, enabling simultaneous regulation of direction and thrust intensity under current disturbance. Therefore, the CPG network can translate high-level decisions into smooth and continuous tail oscillations. This structure provides three main advantages. Firstly, the control approach is designed in two stages to maintain interpretability as a decision-making and oscillatory swimming pattern generator. Secondly, the CPG network ensures continuous oscillatory motion when the high-level command changes rapidly in dynamic situations. Lastly, this approach reduces learning complexity by preventing the DRL agent from directly controlling the nonlinear joint structure. With this integration, the robotic fish swims with an adaptive control structure that enables goal-oriented motion, safe obstacle avoidance, and current-resistant positioning. 

## 3. Results

This section presents the simulation details of the proposed DRL-based policy, including the simulation setup, comparisons, and performance analyses. The simulations are carried out in the MATLAB R2023b environment on a workstation equipped with an Intel Core i9-14900HX CPU, 32 GB RAM, and an NVIDIA GeForce RTX 4080 GPU. The CPG-based two-joint robotic fish model is used with link lengths $L_{1}=0.12$ m and $L_{2}=0.10$. The body length of the robot $L_{b}$ is defined as 0.14 m and the caudal fin is defined together with $L_{2}$. The proposed TD3–CPG controller uses a deterministic actor and twin critics. The actor network consists of two fully connected hidden layers with 128 neurons, followed by ReLU activations and a *tanh* output layer. Each critic utilizes two fully connected hidden layers with 128 neurons and ReLU activations, followed by a scalar Q-value output layer. The mini-batch size, discount factor, and target smoothing factor are set to 256, 0.99, and 5 × 10^−3^, respectively. The actor and critic learning rates are selected as 3 × 10^−4^, and Gaussian target policy smoothing and exploration noise are applied during training.

All DRL agents are trained with a fixed random seed and a maximum training budget of 1000 episodes. The approximate training times are 363 min for TD3–CPG, 430 min for DDPG–CPG, 35 min for PPO–CPG, and 392 min for TD3 without CPG. The proportional (*Kp*), integral (*Ki*), and derivative (*Kd*) gains are selected using a grid search and set to *Kp* = 1.65, *Ki* = 0.07, and *Kd* = 0.25. The selected controller is also evaluated under the same test protocol. The simulations are divided into three challenging tasks. While task 1 involves target reaching in an obstacle-free environment, task 2 involves obstacle avoidance and reaching the target point. Task 3 performs station keeping under various current disturbances.

### 3.1. General Comparison and Evaluation for Target-Reaching Task

**Figure 4:**
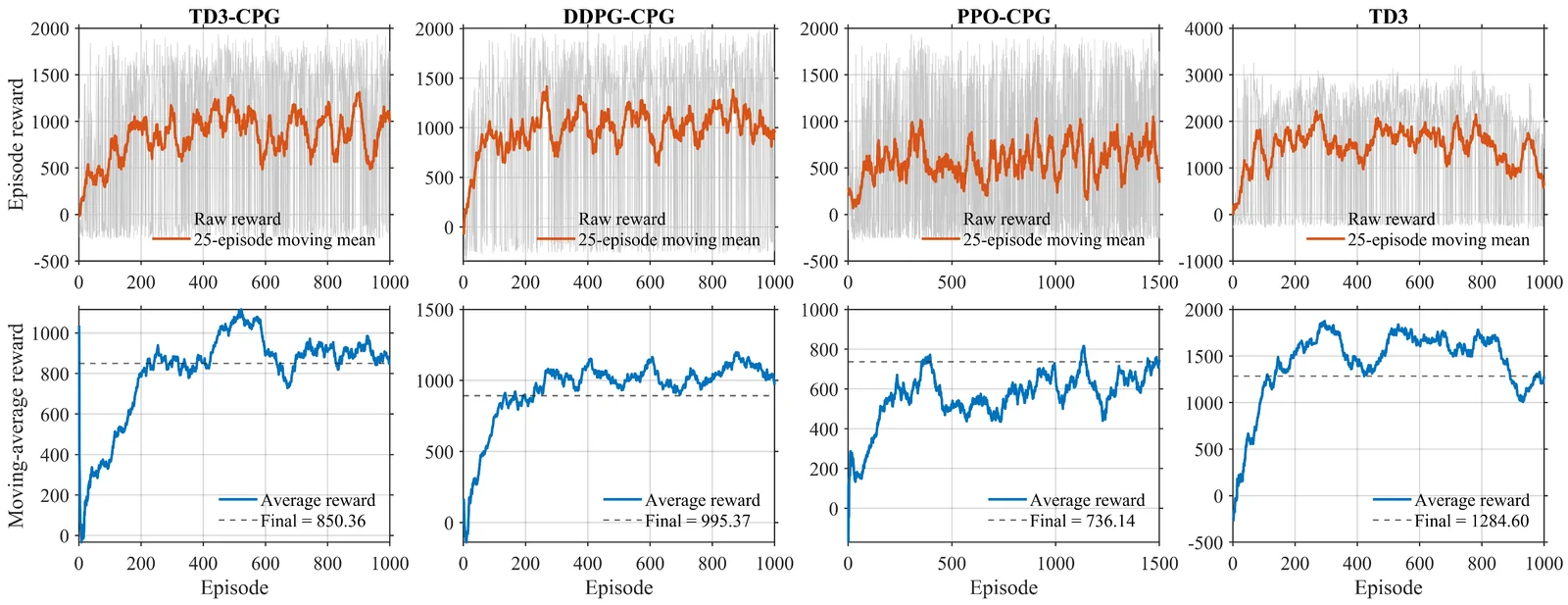
Training processes of the DRL models in target-reaching task.

The proposed TD3–CPG model is compared in detail with DDPG–CPG, PPO–CPG, PID–CPG, and TD3 without CPG for the target-reaching task. The CPG-based DRL models use the same robotic fish dynamics, CPG structure, observation variables, action limits, and evaluation conditions. The comparisons of TD3–CPG, DDPG–CPG, and PPO–CPG present the effect of the DRL algorithm used for high-level CPG modulation. In addition, PID–CPG is included as a conventional control method, while only TD3 evaluation is given to show the effect of the CPG network. Figure 4 shows the training processes of the DRL-based models.

**Figure 5:**
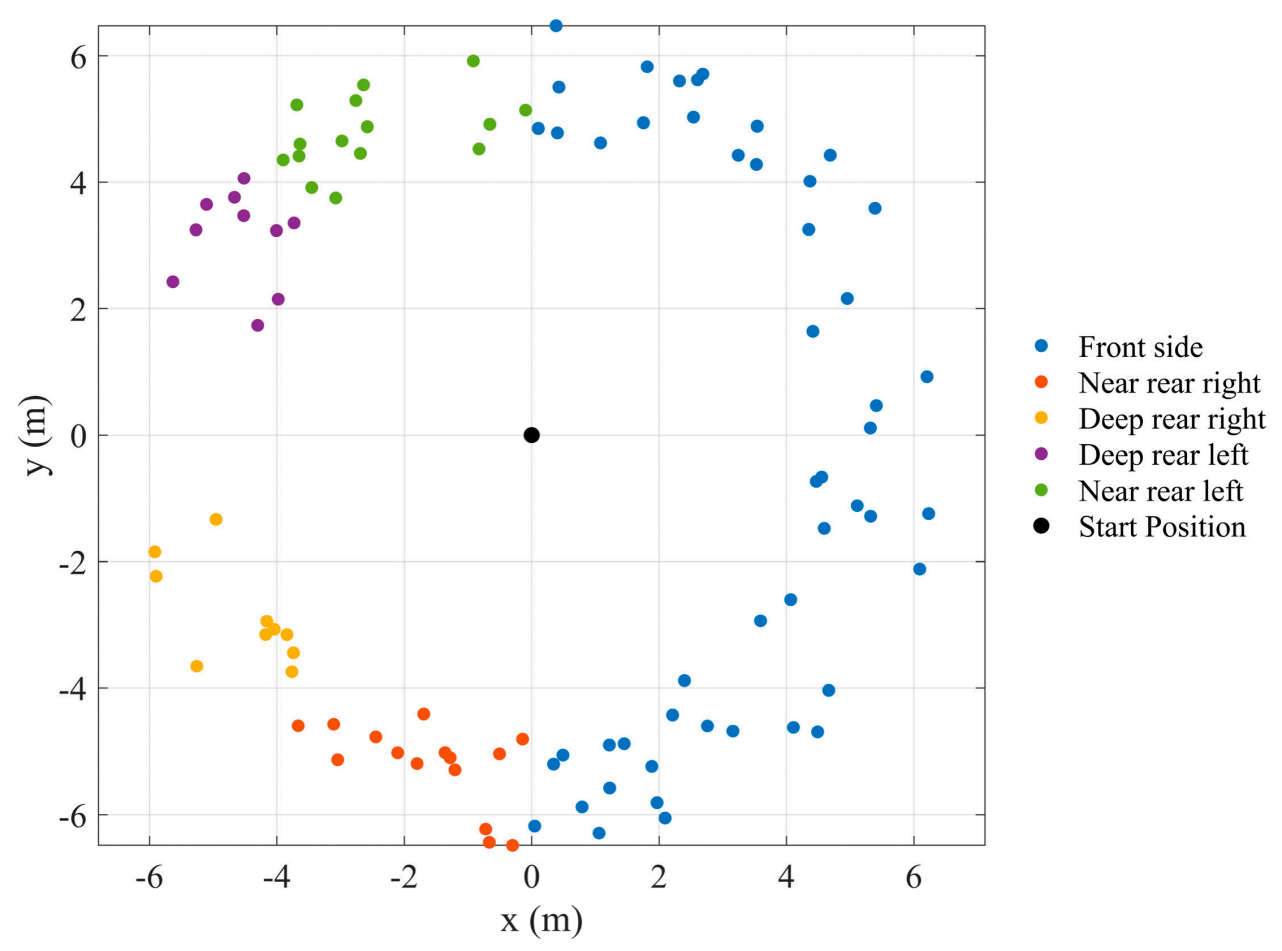
Target distribution.

A comprehensive target-reaching benchmark is designed for the comparisons. As shown in Table 1, the evaluations include 100 target locations distributed across five directional ranges relative to the initial heading of the robot. The target set contains 50 front-side, 30 near-rear, and 20 deep-rear targets. While the front-side targets represent forward and lateral target-reaching cases, the near-rear and deep-rear targets require larger orientation maneuvers to the target. Therefore, the evaluations present the ability of each controller to handle difficult direction changes. Figure 5 shows the target distribution used for all evaluated methods. The fixed start position of the robotic fish is (0,0) and in the positive x-direction. Table 1 shows the target distribution, and the target distance is selected between 4.5 m and 6.5 m, and the target radius is set to 0.15 m. Each method is evaluated in 100 episodes, and the maximum episode duration is 30 s.

Table 2 presents the overall comparison results of the controllers for 100 evaluation episodes. The proposed TD3–CPG model achieves the highest success rate with 100 successful trials out of 100 episodes. The Wilson 95% confidence interval (CI) is [96.30%, 100.00%], and the performance remains statistically reliable. DDPG–CPG reaches 94.0% success with a Wilson interval of [87.52%, 97.22%], while PPO–CPG achieves 77.0% success with a wider CI of [67.85%, 84.16%]. The PID–CPG baseline reaches 89.0% success, and only TD3 reaches 93.0% success. These results show that all methods can produce target-oriented motion, but TD3–CPG provides the most reliable convergence. TD3–CPG reaches the target with a mean arrival time of 14.756 ± 2.633 s. This value is lower than the DDPG–CPG result of 15.570 ± 3.860 s and remains comparable to PPO–CPG and PID–CPG. In terms of path efficiency, TD3–CPG gives a path stretch of 1.139 ± 0.197, while DDPG–CPG gives 1.212 ± 0.318. This indicates that TD3–CPG generates more compact trajectories than DDPG–CPG under the same targets. PPO–CPG has a similar average path stretch to TD3–CPG, but the lower success rate shows that this efficiency is not maintained for all target directions. The energy proxy of TD3–CPG is 1.325 ± 0.237 m^2^/s, which is lower than DDPG–CPG, PPO–CPG, and PID–CPG.

**Figure 6:**
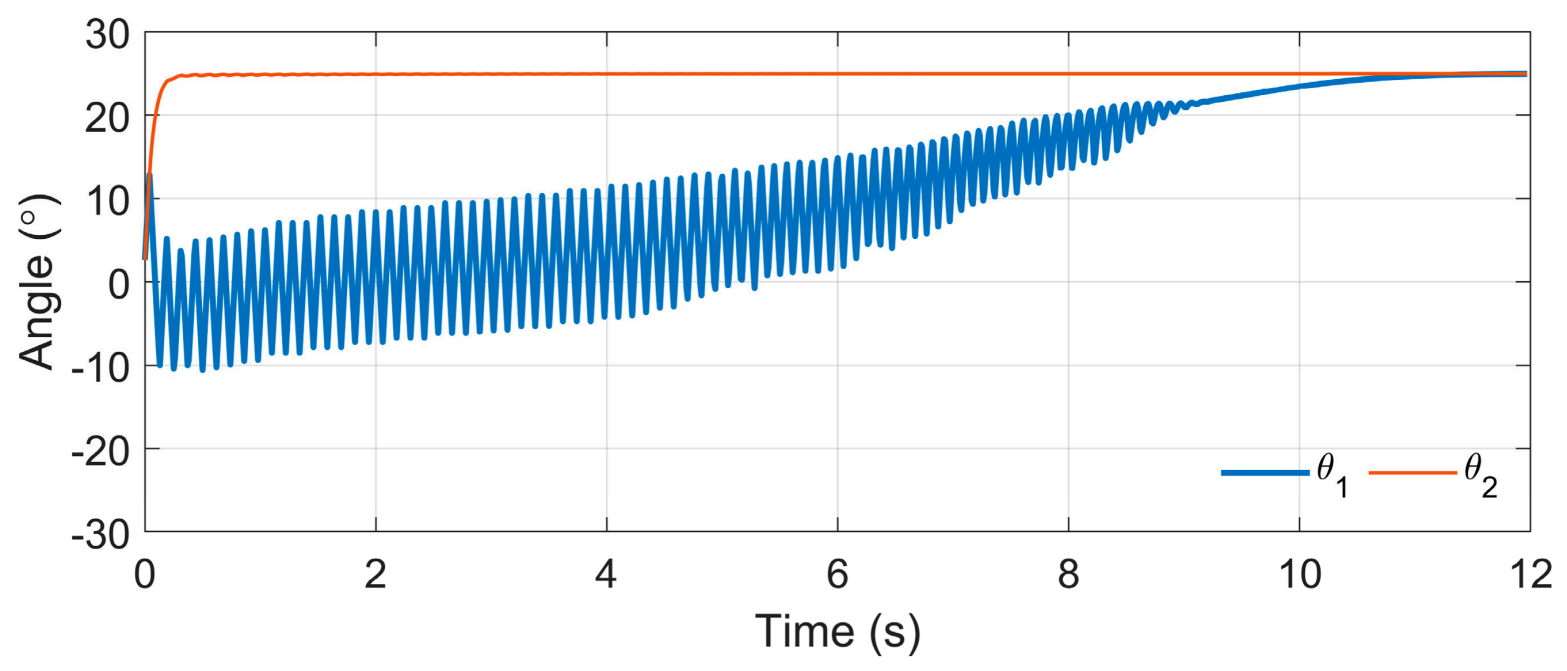
Tail link angles of the best episode generated with only TD3.

The TD3 without CPG method gives a lower path stretch and energy proxy. However, this controller directly generates joint commands without the CPG network. Although only TD3 achieves a relatively high target-reaching success rate of 93.0%, the joint-level locomotion shows that this success is not obtained with a biomimetic swimming pattern. Figure 6 presents the tail link angles of the best episode generated with only TD3. The second link remains close to its angular limit for most of the motion, with a saturation ratio of 92.918 ± 6.716%. The sine-fit coefficients are also very low for both joints, with R^2^ = 0.016 ± 0.016 for *θ*_1_ and R^2^ = 0.001 ± 0.001 for *θ*_2_. As a result, the biomimetic joint-motion rate remains 0.0% despite the high target-reaching success rate. These results show that the TD3 approach without a CPG network can reach the target, but it does not provide the biomimetic swimming patterns. Therefore, the CPG network can effectively generate the rhythmic and applicable swimming patterns for the tail links. When all results are examined, the TD3–CPG provides the most robust target-reaching performance among the compared controllers. While DDPG–CPG achieves a high success rate, this model gives larger arrival time, path stretch, and energy proxy. The PPO–CPG also shows good performance in some successful trials, but it has a lower success rate. PID–CPG has a lower success rate than TD3–CPG. To further analyze the controller performances, target-wise evaluation results are given in Table 3.

According to Table 3, the proposed TD3–CPG model effectively reaches all target types. The arrival time increases from the front-side targets to the deep-rear targets. The mean arrival time is 13.087 ± 1.391 s for front-side targets, while it increases to 18.324 ± 2.151 s and 19.056 ± 1.734 s for the deep rear right and deep rear left targets, respectively. DDPG–CPG performs successfully in most target sections. While it reaches all front-side, near-rear, and deep rear left targets, its performance drops for deep rear right targets, with only 4 out of 10 targets. This result shows that DDPG–CPG has a directional weakness under rear-side target conditions. In addition, arrival times in rear sections are generally longer than TD3–CPG. For example, the average arrival time for deep rear left targets is 22.495 ± 3.060 s for DDPG–CPG, while TD3–CPG reaches the same targets in 19.056 ± 1.734 s. PPO–CPG also gives high performance in the front-side and near rear right targets, but the success rate decreases in the other rear target groups. It reaches 9 out of 15 near rear left targets, 4 out of 10 deep rear right targets, and none of the deep rear left targets. These results show that PPO–CPG is less stable when the target requires a large orientation maneuver.

For the PID–CPG control method, it reaches all front-side targets and provides acceptable performance in near-rear directions. However, its success rate decreases for deep-rear targets, reaching 6 out of 10 deep rear right targets and 7 out of 10 deep rear left targets. TD3 without the CPG model also achieves high success in front-side and near-rear targets, reaching all targets in these regions. However, its success rate decreases to 70.0% for deep rear right targets and 60.0% for deep rear left targets. Although TD3 without CPG can learn target-reaching behavior, removing the CPG network reduces the reliability of the swimming patterns. Based on the distribution of targets, the main advantage of TD3–CPG appears in the rear target directions. While all methods are effective in the front-side workspace, DDPG–CPG, PPO–CPG, PID–CPG, and TD3 show performance degradation in more difficult rear-side targets.

To analyze the performance differences, Table 4 compares the paired success and performance results of the proposed TD3–CPG controller against the baseline controllers. Compared with DDPG–CPG, TD3–CPG succeeds in all episodes, while DDPG–CPG succeeds in 94 episodes. The remaining episodes are completed only by TD3–CPG. The McNemar *p*-value is 0.0412 at the 5% level. In addition, TD3–CPG succeeds in 23 target sections where PPO–CPG fails, achieving a success rate difference of 23.0%, with a McNemar *p*-value of 4.49 × 10^−6^. TD3–CPG also provides higher success than PID–CPG and TD3 without CPG, with success rate differences of 11.0% and 7.0%, respectively. Table 4 also presents the paired differences in the performance metrics. These differences are calculated only for the episodes where both methods successfully reach the target. Compared with DDPG–CPG, TD3–CPG reaches the target 1.118 s faster on average, and the bootstrap 95% CI remains [−1.517, −0.776]. TD3–CPG also gives lower path stretch, shorter path length, and lower energy proxy. A similar trend is observed in the comparison with PPO–CPG. TD3–CPG reduces the arrival time by 0.909 s on average, and the bootstrap 95% CI is [−1.374, −0.534]. The path stretch, path length, and energy proxy differences are also negative, indicating that TD3–CPG produces more efficient trajectories than PPO–CPG in the paired successful trials.

Compared with PID–CPG, TD3–CPG provides a higher success rate and slightly lower performance metrics in the paired successful episodes. The path stretch and path length differences are negative, which indicates more compact target-reaching trajectories. The arrival time and energy proxy differences are small, but the success comparison shows that the learned TD3–CPG modulation is more reliable than the PID under target directions. TD3 also reaches many targets. However, the TD3–CPG reaches the common successful targets 2.113 s faster on average than TD3 without CPG. On the other hand, TD3 gives lower path stretch, path length, and energy proxy values in some paired trials. TD3 also uses high-frequency, saturated, and non-sinusoidal joint motions. Therefore, the CPG component is important not only for target-reaching success but also for preserving rhythmic swimming kinematics.

According to the comparative results, TD3–CPG provides the most reliable and effective control performance among all methods. It achieves complete success in the target-reaching benchmark, maintains directional robustness in all targets, and preserves rhythmic CPG-based swimming. Therefore, TD3–CPG is selected as the control structure for the following task evaluations.

### 3.2. Task 1: Target Reaching Results

**Figure 7:**
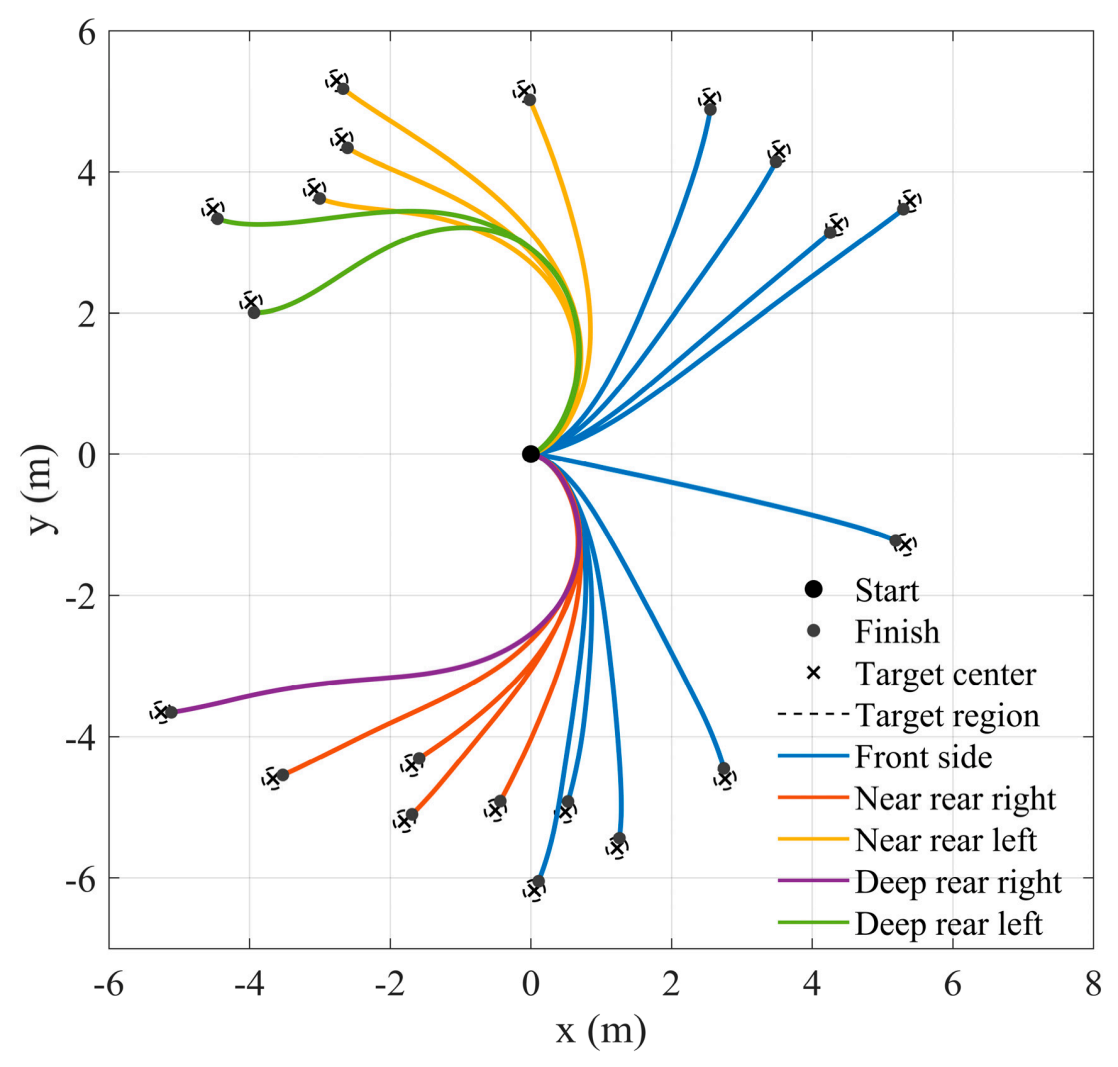
Robotic fish trajectories for target reaching over 20 evaluations. Different colors represent the target locations.

In task 1, the target-reaching behavior of the designed TD3-CPG model is further examined in terms of trajectory formation, convergence behavior, and locomotor response. Therefore, representative trajectories are used to evaluate the behavior of the robotic fish during the task. In Figure 7, 20 episodes are randomly selected, and these episodes include front side, near rear, and deep rear target directions. In this evaluation, all trajectories reach the target region. These targets cover a distance range of 4.52–6.47 m and a direction range from −145.19° to +151.63°. The mean arrival time is 14.525 ± 2.118 s, and the mean path stretch is 1.135 ± 0.180. The TD3–CPG produces smooth and target-oriented trajectories for different targets. For front-side targets, the robotic fish generally follows compact paths with limited lateral deviation. These trajectories are almost aligned with the target direction and represent the forward swimming. For near rear and deep rear targets, the robotic fish generates a curved orientation motion and then swims to the target region. These results show that the TD3 policy effectively modulates the λ, and the CPG network maintains rhythmic undulatory motion during both turning and forward swimming phases. Although the rear target trajectories are longer and more curved than the front side trajectories, the robotic fish reaches the target without unstable oscillations. Therefore, the designed model can maintain stable locomotion while adapting the swimming direction according to the target location.

**Figure 8:**
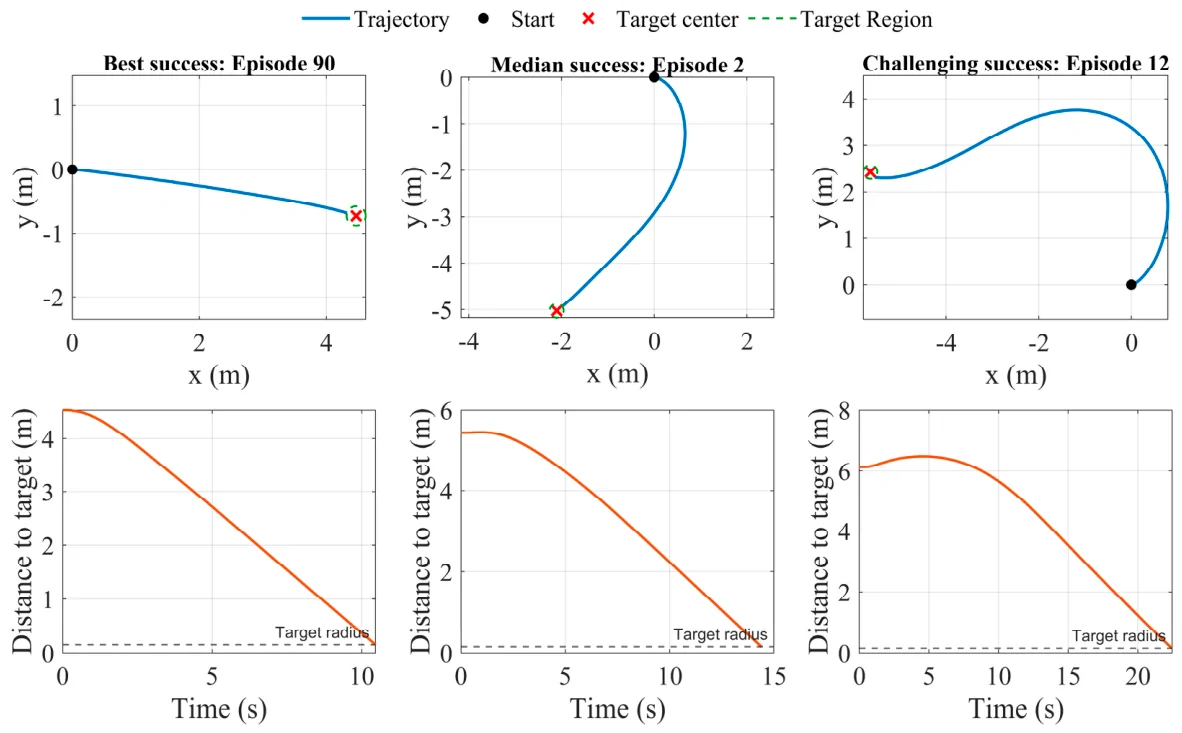
Representative TD3–CPG target reaching episodes and distance to target responses.

To further clarify the behavioral structure of task 1, Figure 8 presents the representative target reaching episodes selected from the evaluations. These cases include the best, the median, and rear direction successful episodes. In the best episode, the target is located in front of the robot. The robotic fish reaches the target in 10.46 s with a path length of 4.382 m and a path stretch of 0.968. The trajectory is almost direct, and the distance of the target response decreases smoothly. The median successful episode is achieved with the near rear right target task. In this case, the robotic fish performs a curved orientation and then swims to the target. The arrival time is 14.41 s, the path length is 6.181 m, and the path stretch is 1.135. The challenging episode represents a deep rear left target, which requires a larger orientation maneuver. The robotic fish reaches the target in 22.47 s with a path length of 9.807 m and a path stretch of 1.599. The trajectory includes an initial wide turning phase, followed by forward swimming to the target. Therefore, the representative episodes show that the proposed controller adapts the trajectory according to the target direction. The TD3 policy can effectively modulate the CPG to generate both direct target reaching and large orientation maneuvers.

**Figure 9:**
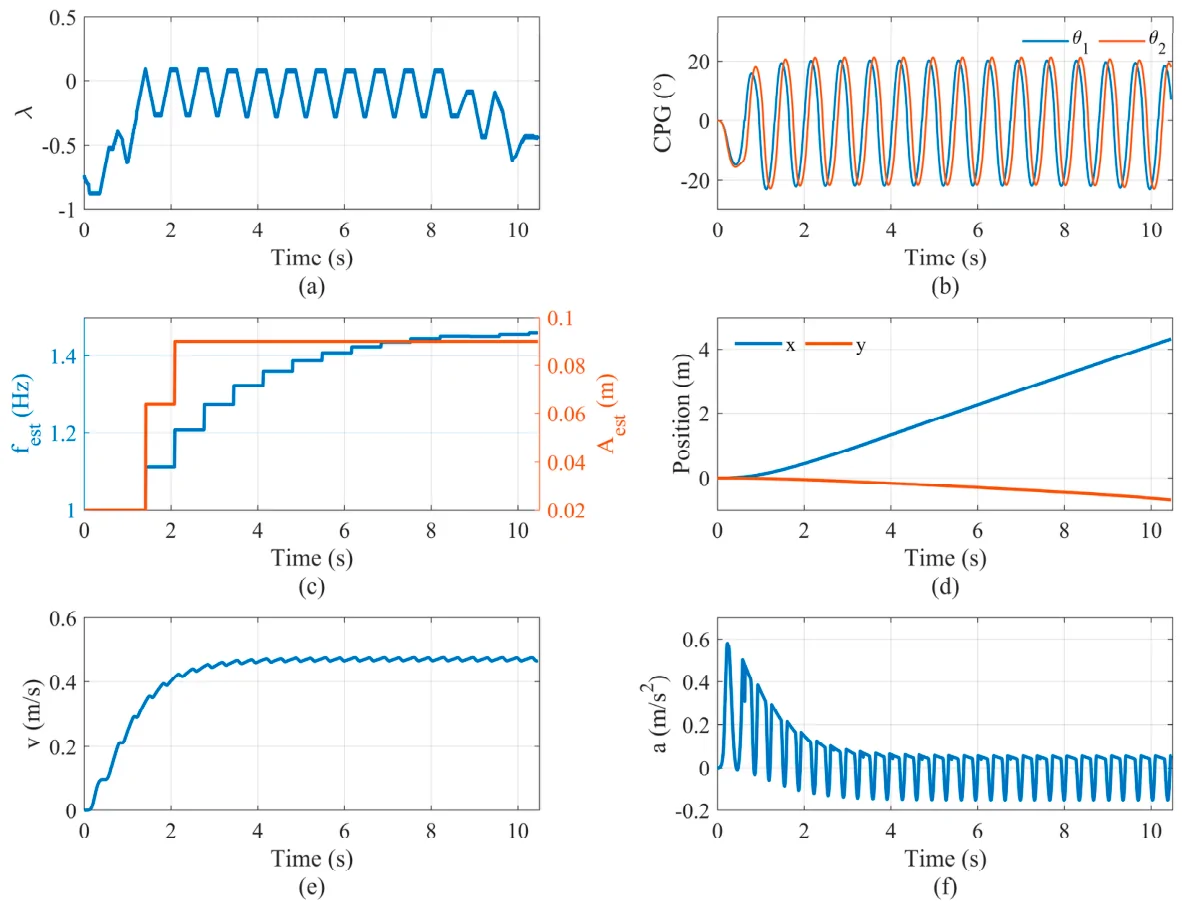
Control and locomotor results of the robotic fish for the most successful episode. (**a**) Evolution of the λ. (**b**) CPG outputs, (**c**) estimated oscillation frequency and oscillation amplitude, (**d**) position, (**e**) swimming speed, (**f**) acceleration.

Figure 9 shows the CPG and kinematic responses for the most successful target reaching episode. In this episode, the robotic fish reaches the target in 10.46 s with a smooth trajectory. The λ remains bounded during the motion. It first gives a negative value to provide rapid swimming motion to the target direction and then oscillates around 0. The CPG outputs $\left(\theta_{1},\theta_{2}\right)$ preserve smooth and oscillatory behavior during the episode. The joint angles remain within approximately ±23°. The DRL policy modulates the CPG and does not directly control the joint angles. Therefore, the rhythmic locomotion pattern is maintained by the CPG structure while the TD3 policy adjusts the swimming direction. The estimated oscillation frequency increases from 1 Hz to approximately 1.46 Hz during the episode. Furthermore, the estimated amplitude reaches 0.09 m. The CPG generates a faster oscillation pattern after the transient phase, and the robotic fish reaches stable swimming while maintaining target-directed motion. The horizontal position of the robot increases almost monotonically, while the vertical position changes to the negative direction. The swimming speed obtained is 0.47 m/s. The acceleration response is higher during the initial turning and then decreases to 0. As a result, the TD3 policy provides effective modulation through λ, while the CPG network preserves stable oscillatory outputs. This coordination enables smooth target-directed swimming with the rhythmic motion pattern. To examine whether the trained TD3–CPG policy depends on the nominal initial pose, a target-reaching evaluation is also conducted under randomized initial conditions. The initial *x* and *y* coordinates are independently sampled from uniform distributions over [−2, 2] m, while the initial heading is sampled over the [−180°, 180°] range. The target is fixed at (5.5, 0) m, with a target radius of 0.15 m and a maximum episode duration of 30 s. A fixed target position is also selected as (5.5, 0.0) m. The same trained TD3–CPG policy is used in all trials without retraining or fine-tuning. Table 5 presents the randomized initial-state robustness of the TD3–CPG controller.

According to Table 5, the proposed controller successfully reaches the target in 49 of the 50 randomized trials, corresponding to a success rate of 98.0%. The Wilson 95% CI is [89.50%, 99.65%]. The lower confidence bound remains at 89.50%, indicating that the observed high success rate is maintained after accounting for the finite number of evaluation trials. Across the 49 successful trials, the mean arrival time is 14.574 ± 3.489 s, with a 95% CI of [13.572, 15.576] s. The only unsuccessful trial starts from (−1.807, 1.990) m with an initial heading of 160.86°, representing a challenging combination of large positional displacement and a nearly opposite initial orientation. Therefore, these results indicate that the TD3–CPG policy is not restricted to the nominal initial pose and maintains reliable target-reaching behavior across a broad range of previously unseen initial conditions.

### 3.3. Task 2: Obstacle Avoidance in Target Reaching

To avoid obstacles in target reaching, the same dynamic model and TD3-CPG structure are used, but the swimming environment is transformed into an obstacle-constrained navigation task. The episode duration and integration step are selected as 30 s and 0.01 s. The obstacle is designed as circular and with a radius from 0.25 to 0.50 m. In addition, rectangular obstacles are used to analyze the robustness of the control structure. Obstacle detection is performed with an 11-beam range configuration covering 180° with sensor noise and a detection range of 4.0 m. Radial range measurements, minimum obstacle clearance, left and right obstacle asymmetry, relative orientation of the nearest obstacle, and normalized clearance information are added to the observation vector. The control action is the λ for orientation. Table 6 presents the quantitative performance results of the proposed TD3–CPG controller for obstacle avoidance in target reaching.

According to Table 6, the proposed controller solves the obstacle avoidance and target-reaching problem with a high level of reliability. In 100 evaluation episodes, the robotic fish achieves a success rate of 98.0%, with a Wilson 95% CI of [93.00%, 99.45%]. The collision rate remains at 2.0%, with a CI of [0.55%, 7.00%]. The safe-pass rate is also 98.0%, with the CI of [93.00%, 99.45%]. These values show that the controller can simultaneously perform target convergence and collision-free navigation with statistically reliable performance. The overlap between the success rate and the safe-pass rate shows that the controller can overcome obstacles with a safe distance, rather than merely lightly touching the obstacle boundaries. The temporal efficiency of successful trajectories remains high. The average arrival time is 13.884 ± 1.386 s, showing that obstacle avoidance introduces only a moderate temporal cost while maintaining consistent convergence behavior. This result is also supported by the path stretch. The controller gives an average path stretch of 1.077 ± 0.041 over all episodes and successful episodes. Therefore, the controller does not avoid obstacles by producing unnecessarily long trajectories. Instead, it finds only the necessary direct path to the target to maintain a safe distance. The obtained results show robust performance, and the controller maintains both path economy and collision avoidance within the same policy.

The clearance results also confirm the safety-related performance. The minimum clearance is 0.551 ± 0.204 m over all episodes and 0.562 ± 0.192 m in successful episodes. In contrast, the failed episodes have a minimum clearance of 0.048 m. The safety criterion is reflected in the evaluation results. These values are significantly greater than the collision threshold, and it can be shown that successful navigation maintains a meaningful spatial path around obstacles, rather than traversing narrow and unsafe margins. The average speed remains 0.422 ± 0.009 m/s over all episodes and 0.422 ± 0.008 m/s in successful episodes. These values indicate that the controller avoids obstacles without producing slow swimming behavior. The failed episodes have a lower average speed of 0.395 ± 0.014 m/s, suggesting that these cases are related to insufficient geometric avoidance. A similar result is observed in the energy proxy. The energy proxy is 2.397 ± 0.014 m^2^/s over all episodes and 2.398 ± 0.013 m^2^/s in successful episodes, while it decreases to 2.357 ± 0.006 m^2^/s in failed episodes. The mean λ is 1.633 ± 0.459 over all episodes and 1.635 ± 0.453 in successful episodes. Compared to task 1, this increased orientation requires continuous orientation to target tracking to ensure obstacle avoidance. In the failed episodes, the mean λ is 1.544 ± 1.002. The collision results indicate that the controller applies a significant orientation effort but cannot convert it into a collision-free maneuver. According to the obtained results, task 2 achieves a highly favorable balance between target-reaching performance, collision avoidance, and trajectory efficiency. The controller maintains a nearly safe path stretch of 1.077 ± 0.041 in successful trials while maintaining a 98.0% success rate. The proposed architecture produces integrated motion behavior where safety constraints are integrated with only limited temporal and geometric costs.

**Figure 10:**
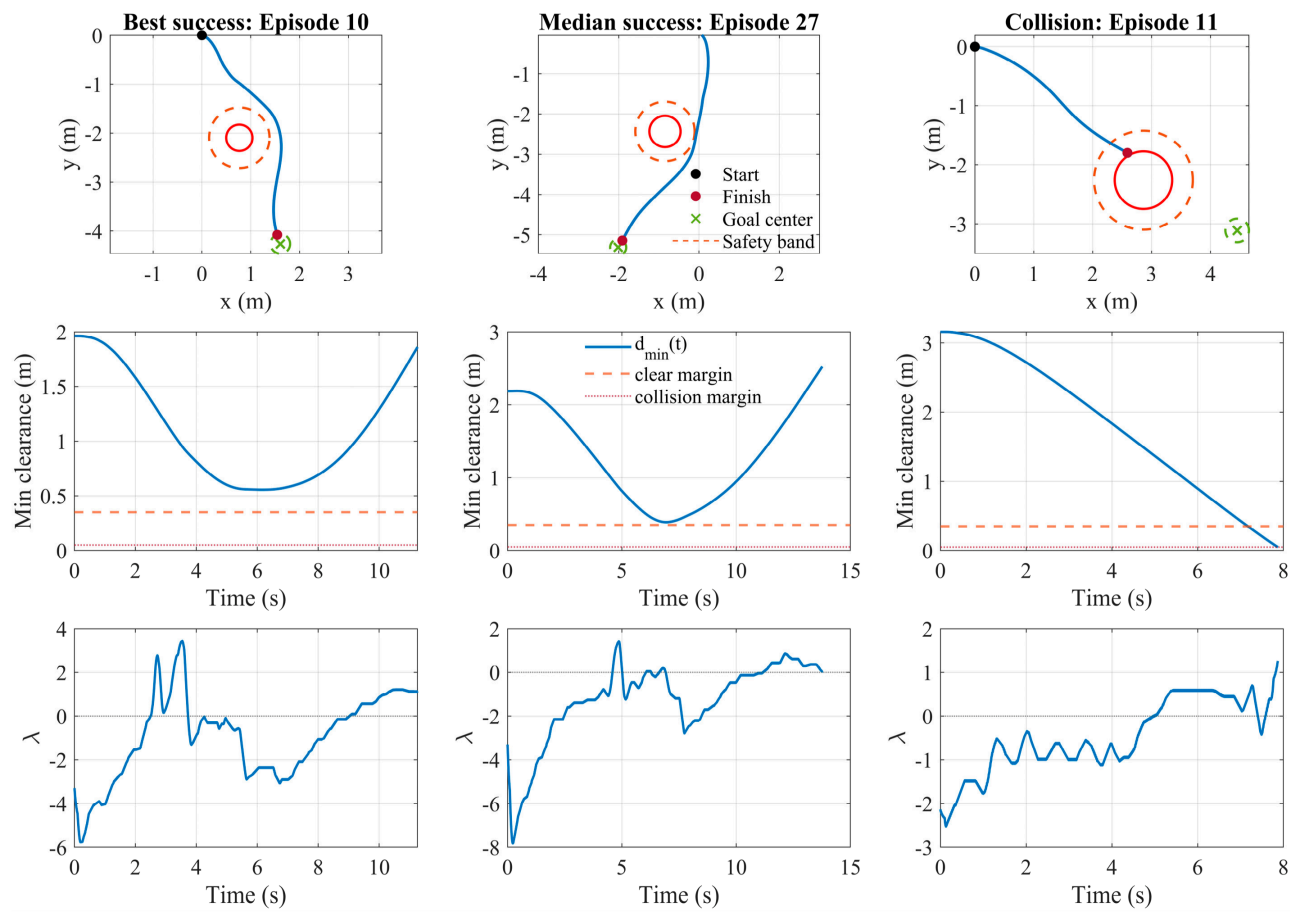
Obstacle avoidance performance for task 2.

Figure 10 presents the obstacle avoidance behavior of the TD3–CPG controller for task 2. The obtained results include three representative episodes: the most successful, the median successful, and the collision episodes, respectively. The top row shows the robot trajectories, the middle row gives the minimum obstacle clearance ($d_{min}$), and the bottom row presents the temporal evolution of the λ. In this figure, the geometric efficiency, safety preservation, and orientation characteristics of the proposed controller in successful and failed obstacle avoidance scenarios are detailed.

In the most successful episode, the robotic fish follows a compact trajectory and passes the circular obstacle with limited lateral deviation before reaching the target. The clearance decreases during the approach to the obstacle, remains above the safety margin, and then increases after the obstacle is passed. This behavior shows that the controller can preserve a safe distance without an unnecessary path. In the median successful episode, the trajectory is longer and includes a wider avoidance maneuver. However, the minimum clearance also remains above the collision margin, and the robotic fish reaches the target. The corresponding Wilson 95% for the success and safe-pass rates is [93.00%, 99.45%]. The clearance results also show the safety performance of the controller. In successful episodes, the minimum clearance is 0.562 ± 0.192 m, which indicates that the robot generally maintains a meaningful spatial margin around obstacles. In contrast, the collision episode shows a decrease, and the clearance falls below the collision margin. The failed episodes have a minimum clearance of 0.048 m. λ represents how the TD3 policy modulates the CPG during obstacle avoidance. In successful episodes, λ changes before the nearest obstacle interaction, allowing the robot to swim toward the target. The most successful episode uses a high modulation response, while in the collision, the λ command also changes, but sufficient maneuverability cannot be produced. Therefore, failures depend on both the magnitude of the λ and the rapid modulation response. The TD3–CPG can integrate obstacle avoidance and target-reaching behavior within the same CPG modulation policy. These results show that the controller is effective in generating efficient paths during obstacle-constrained tasks with high reliability and a low collision rate.

**Figure 11:**
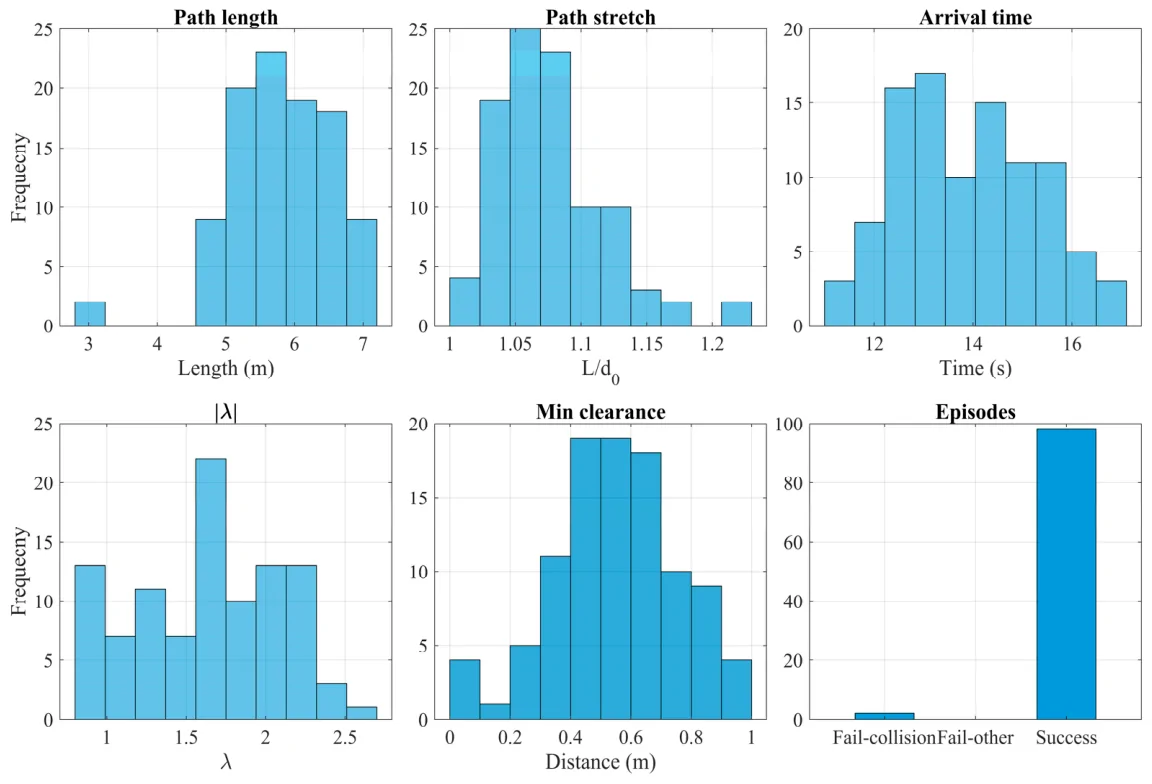
Statistical distributions of obstacle avoidance performance in task 2.

Figure 11 presents the statistical distributions of obstacle avoidance performance in the target-reaching task. The histograms summarize the path length, path stretch, arrival time, mean λ, and minimum obstacle clearance over 100 evaluation episodes. The episode distribution is also given to show the number of successful and collision cases. The path length distribution is generally concentrated between 5.0 m and 7.0 m, allowing the robotic fish to avoid obstacles without generating long trajectories. Path stretch values are mainly distributed between 1.03 and 1.10. Therefore, the proposed controller maintains geometric efficiency while sufficiently modifying the trajectory to avoid obstacles.

The arrival time distribution shows that most successful episodes are completed between approximately 12 s and 16 s. Therefore, obstacle avoidance can occur without significant delays in reaching the target. According to the mean λ distribution, different levels of CPG modulation can be achieved depending on the obstacle geometry and target orientation, and the mean λ value is 1.633 ± 0.459 in all episodes. Considering the minimum clearance, it is clear that the controller ensures safe behavior, and a few trajectories are close to the collision boundary. The mean minimum clearance of successful episodes is 0.562 ± 0.192 m, while the clearance of failed cases is much smaller at 0.048 m. This difference indicates that successful navigation maintains a significant safety margin around the obstacles. The robotic fish effectively performs 98 out of 100 episodes, with only two episodes resulting in a collision.

**Figure 12:**
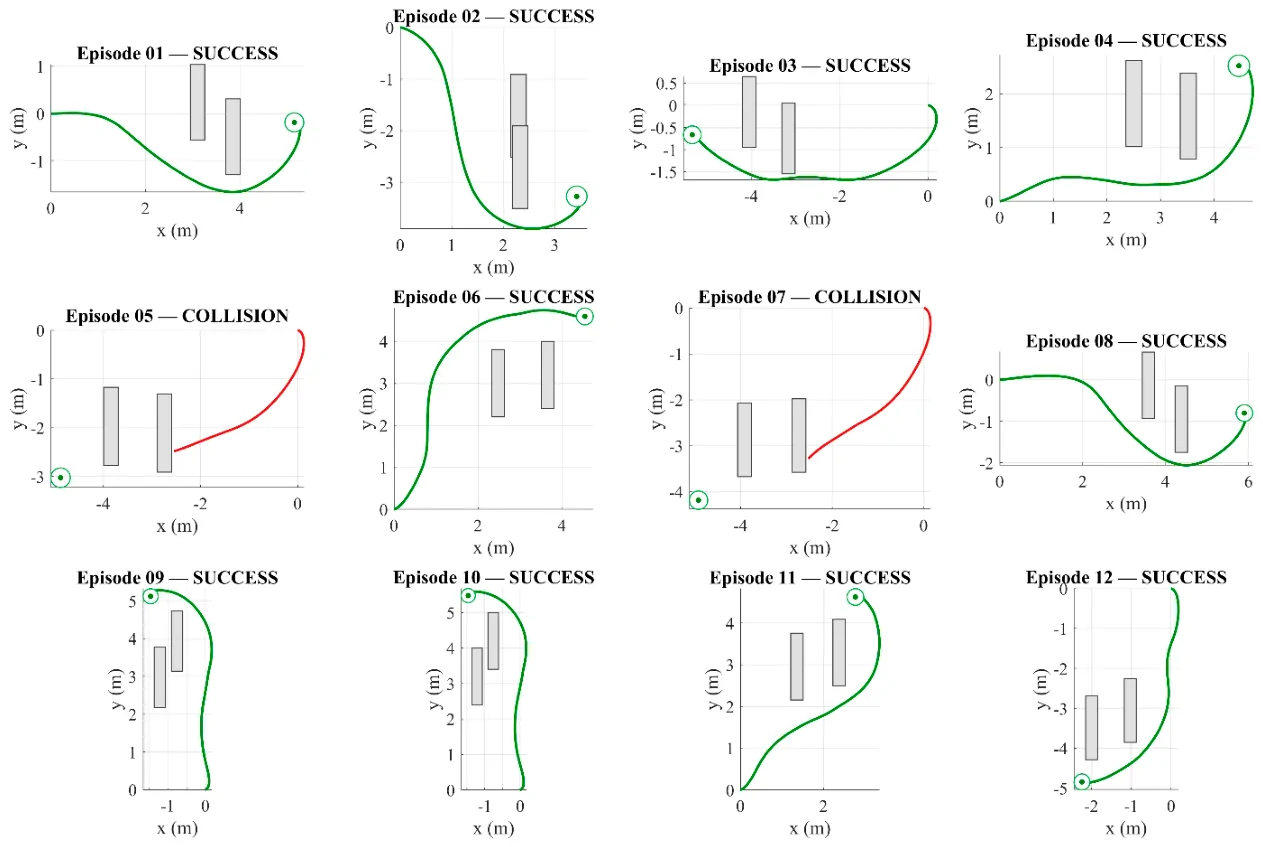
Multi-episode visualization with multiple rectangular obstacles for scenario 6.

Table 7 presents the scenario-wise quantitative performance of the controller in rectangular obstacle configurations. Each scenario is evaluated over 12 trials. The task success, collision frequency, temporal convergence statistics, path efficiency, and minimum clearance levels under different obstacle geometries are evaluated. Figure 12 also shows the episode visualizations with multiple rectangular obstacles. According to Table 7, eight scenarios and a total of 96 trials are performed with 88 successful operations, achieving an overall success rate of 91.7%. The total number of collision cases is limited to seven, corresponding to a collision rate of 7.3%. These values show that the controller generally preserves target-directed motion even in challenging geometries, and the failure cases remain around obstacle interaction rather than full navigation disruption. The success rates of the scenarios are obtained between 83.3% and 100.0%. Specifically, scenarios 3 and 5 achieve 12/12 success with no collisions, showing the reliability of the controller in the rectangular multiple obstacles. The most challenging scenarios are scenarios 1 and 6, each with 10/12 success and 2/12 collisions. However, the controller maintains a successful operation rate in these obstacle configurations. When the arrival time results are analyzed, $T_{reach,50}\;$ changes between 14.01 s and 15.63 s, while the median value achieved is 14.80 s. Similarly, $T_{reach,90}\;$ changes between 16.09 s and 17.26 s, with a general scenario average of 16.67 s. The obstacle avoidance introduces only a limited temporal overhead. Therefore, the controller determines efficient motion patterns, and it completes the task within a suitable and sufficient range. Path efficiency values are also favorable. Scenario-based path stretch remains in the range of 1.11–1.20, with a median value of 1.15 across all scenarios. This is a limited value, and the controller efficiently generates the necessary orientation for a safe pass. When examining the minimum clearance values, these values change between 0.237 m and 0.358 m, with a median value of 0.299 m. For scenario 7, there is also one timeout failure. These values indicate that the robotic fish generally passes obstacles with a proper buffer. In addition, the best-performing scenarios do not include the largest minimum clearance. For example, scenario 3 achieves 100% success and a 0% collision rate with a minimum clearance of 0.237 m, producing a highly satisfactory value for the controller. Therefore, the controller can efficiently utilize the available space while maintaining a safe pass. As a result, the proposed controller can effectively overcome multiple rectangular obstacles with a low collision frequency, stable convergence times, limited path elongation, and significant distance preservation. Furthermore, integrated motion behavior is achieved with safe-pass and target convergence within the same control policy.

For these results, the overall success rate is (88/96 = 91.7%), with a Wilson 95% CI of [84.41%, 95.72%]. The overall collision rate is (7/96 = 7.3%), with a Wilson 95% CI of [3.58%, 14.29%]. One additional trial corresponds to a non-collision failure/timeout, giving a rate of (1/96 = 1.0%), with a Wilson 95% CI of [0.18%, 5.67%]. To examine performance as a function of task difficulty, the rectangular obstacle scenarios are also grouped according to the gap width. For the narrower gap condition, the success rate is (43/48 = 89.6%), and the collision rate is (5/48 = 10.4%). For the wider gap condition, the success rate is (45/48 = 93.8%), and the collision rate is (2/48 = 4.2%). These results indicate that narrower areas exhibit a higher observed collision rate, while the controller still maintains a high overall success rate.

In Figure 12, the green trajectories define the successful target reaching, while red trajectories denote collision cases. The gray bars represent the obstacles. In this scenario, 10 out of 12 episodes are successful, while collisions occurred in two episodes. These results demonstrate that the controller generates adaptable trajectories for various obstacle locations. For multiple and large obstacles, the controller generally generates the correct geometric avoidance actions. From a motion perspective, the orientation actions are structured, repeatable, and target-oriented. Therefore, it can be seen that the controller can efficiently overcome obstacles for single and multiple obstacles.

### 3.4. Task 3: Station Keeping Under Current Disturbances

The main objective of Task 3 is to ensure the robotic fish remains within a target-centered area under external current disturbances. In this task, the robotic fish attempts to compensate for current-induced drift while maintaining stable oscillatory swimming. The same TD3–CPG control structure is used, and the task objective is selected as station keeping. While the DRL policy modulates the CPG outputs, the CPG network maintains rhythmic joint angles. Therefore, station keeping is achieved through the regulation of body orientation and oscillatory swimming motion. Two current types are defined as constant and gust. The constant current type represents a continuous and spatially homogeneous disturbance with fixed magnitude and direction, while the gust current represents a transient pulse-like current disturbance. The current disturbance parameters for the station keeping evaluations are given in Table 8. Here, the current speed range, direction offset, and temporal profile of currents are detailed. In the constant current, the disturbance acts during the whole episode, and the current speed is randomly selected between 0.25 m/s and 0.35 m/s for each episode. In the gust current, the disturbance is applied as two short-duration Gaussian pulse-type currents. This current has a speed of 0.29–0.31 m/s, but the pulse modulation increases the instantaneous peak speed to approximately 0.494–0.588 m/s. Therefore, the station keeping task is evaluated with both persistent moderate current disturbances and short-duration, high-intensity disturbances. The station keeping region is defined as a circle centered at the desired position with a radius of 0.25 m. The performance results are measured for each current model over 100 evaluation episodes in terms of the stay ratio, mean target distance $\left(d_{mean}\right)$, mean swimming speed $\left(v_{mean}\right)$, mean $\left(\lambda_{mean}\right)$, and estimated CPG frequency $\left(f_{mean}\right)$. Table 9 presents the station keeping statistics obtained from the evaluations. The stay ratio is given with a bootstrap of 95%, while the remaining metrics are given as standard deviation.

According to Table 9, the proposed controller maintains effective station keeping under both disturbance conditions. Under constant current, the robotic fish remains inside the station keeping region for 87.57 ± 12.81% of the evaluation time, with a bootstrap 95% CI of [85.06%, 90.02%]. The mean distance to the target center is 0.155 ± 0.032 m, which remains below the 0.25 m station keeping radius. This result indicates that the controller can compensate for a drift force and keep the robotic fish close to the desired position. Under gust current, the stay ratio increases to 96.88 ± 10.79%, with a bootstrap 95% CI of [94.61%, 98.75%]. The mean target distance is 0.134 ± 0.041 m, which is also below the station keeping radius. Although gust current provides transient disturbances, the controller recovers these perturbations and returns the robot to the target region in most episodes. The higher stay ratio under gust current shows that the gust type disturbance is compensated more effectively than the continuous drift produced by constant current. Under constant current, the mean swimming speed is 0.161 ± 0.031 m/s, while the mean λ is 1.847 ± 0.537. Therefore, the controller requires higher modulation performance to resist the constant current. Under gust current, the mean swimming speed decreases to 0.142 ± 0.014 m/s, and the mean absolute λ to 1.238 ± 0.308. The estimated CPG frequency remains within a stable range in both cases. The mean estimated frequency is 1.389 ± 0.075 Hz under constant current and 1.448 ± 0.059 Hz under gust current. The CPG network maintains rhythmic oscillation, and the TD3 policy effectively modulates the network for station keeping. Overall, it confirms that the proposed controller performs not only in target reaching and obstacle avoidance tasks, but also in station keeping scenarios exposed to current, maintaining stable oscillatory swimming dynamics.

**Figure 13:**
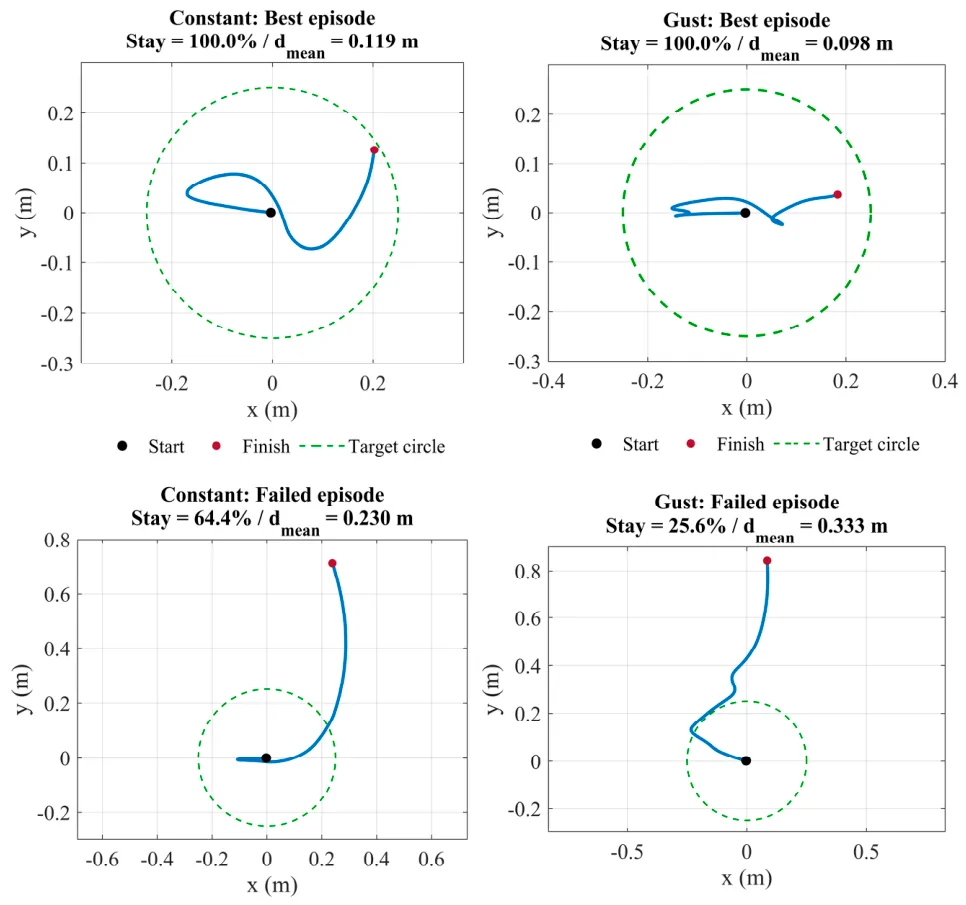
Best and challenging results under constant and gust currents.

Figure 13 shows the station keeping behavior of the robotic fish under constant and gust current disturbances. In this figure, the best and low-performance episodes are given. The yellow circle denotes the station keeping region with a radius of 0.25 m centered at the target. With the best constant current, the robotic fish fully remains in the region with a stay ratio of 100.0% and a mean target distance of 0.119 m. The robot trajectory forms a compact loop around the target center and remains within the station keeping boundary. A similar pattern is observed in the best gust current, where the stay ratio is also 100.0%, while the mean target distance further decreases to 0.098 m. In the failed constant current case, the stay ratio decreases to 64.4%, and the mean target distance increases to 0.230 m. In the gust current case, the degradation is more pronounced. The stay ratio decreases to 25.6%, and the mean target distance increases to 0.333 m, which exceeds the station keeping radius. As a result, the proposed control architecture has two main characteristics. Firstly, successful station keeping is not realized through static behavior. Instead, it results from the fish continuously swimming within the external current, from limited oscillatory motion around the target. Secondly, the difference between best and challenging episodes is mainly reflected in the spatial extent of the drift recovery cycle. In the best cases, the recovery motion remains within the circular boundary, while in the challenging cases, the trajectory expands toward and beyond the allowed region before being redirected. Overall, the representative trajectories illustrate bounded drift-recovery behavior under the selected steady and transient currents.

**Figure 14:**
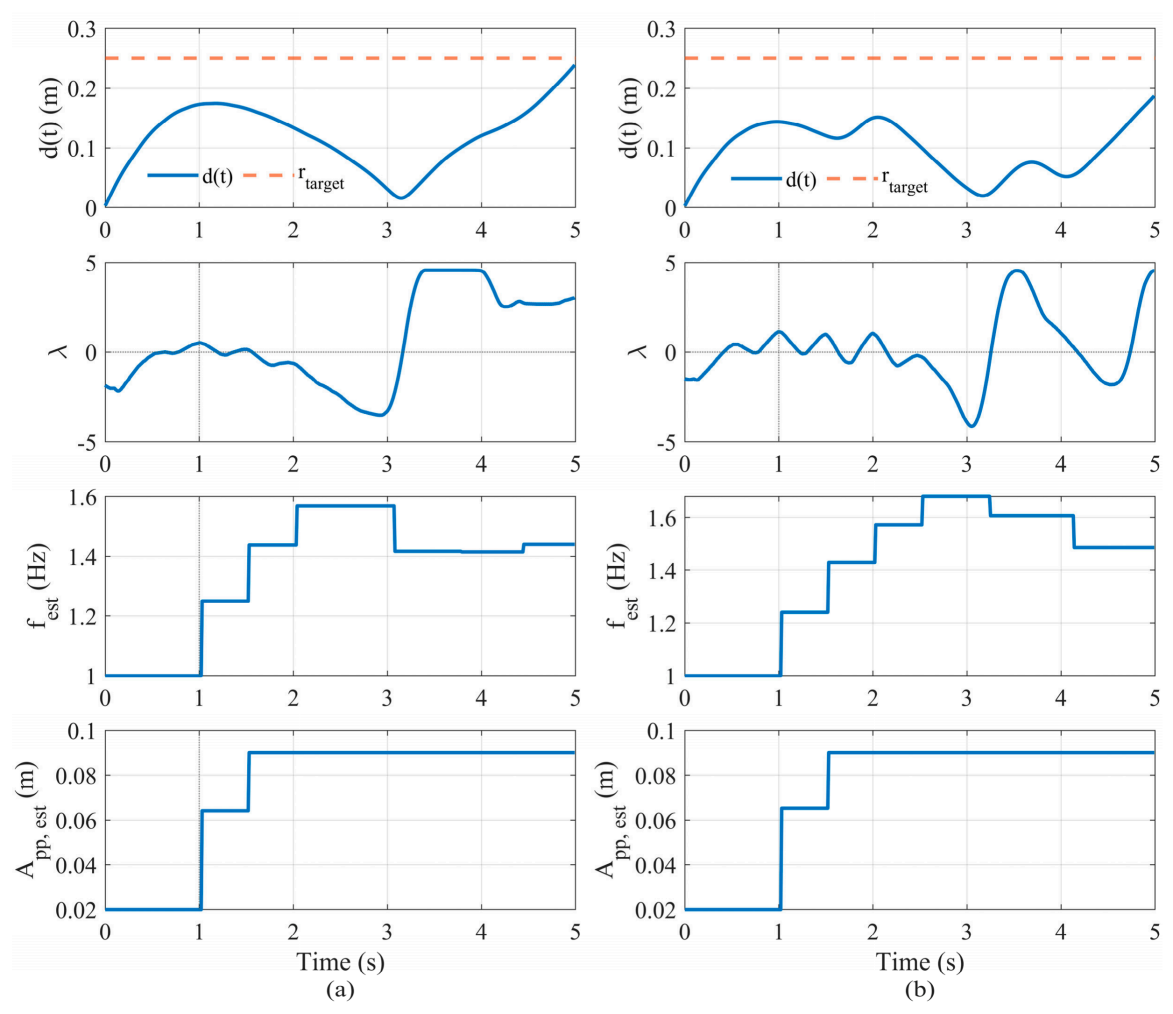
Representative station keeping results under (**a**) constant and (**b**) gust currents.

Figure 14 presents the representative station keeping responses under constant and gust current disturbances. Figure 14a shows the constant current condition, while Figure 14b shows the gust current. In both cases, the distance to the target center remains below the station keeping boundary of 0.25 m during the given episode. In the constant current case, d(t) initially increases from nearly 0 m to approximately 0.17 m due to the current. After this transient, the controller generates a corrective response and reduces the distance to nearly 0.01 m around 3 s. Later, the distance rises again and remains below the 0.25 m boundary. In the gust current, the distance response is bounded inside the station keeping region. The maximum distance remains around 0.18 m, which is lower than 0.25 m. Compared with the constant current, the gust current shows smaller distance variation after the initial response. In the constant current case, λ varies approximately between −3.5 and +4.5, with a strong positive correction after 3 s. This indicates that stronger modulation is required to resist the persistent drift. In the gust current, λ also changes within a bounded range and shows transient correction phases following the disturbance effect. The estimated CPG frequency and oscillation amplitude further explain the locomotor adaptation. In both current conditions, f increases from 1.0 Hz during the initial phase to 1.56 Hz during compensation. After the transient phase, the frequency settles around 1.43 Hz. Similarly, estimated oscillation amplitude increases rapidly from 0.02 m to its upper value of 0.09 m. These changes show that the CPG network increases rhythmic propulsion during the station keeping response while preserving stable oscillatory behavior. As a result, the proposed architecture provides coordinated, limited directional correction and adaptive rhythmic activation to maintain position constraint under different current disturbances.

**Figure 15:**
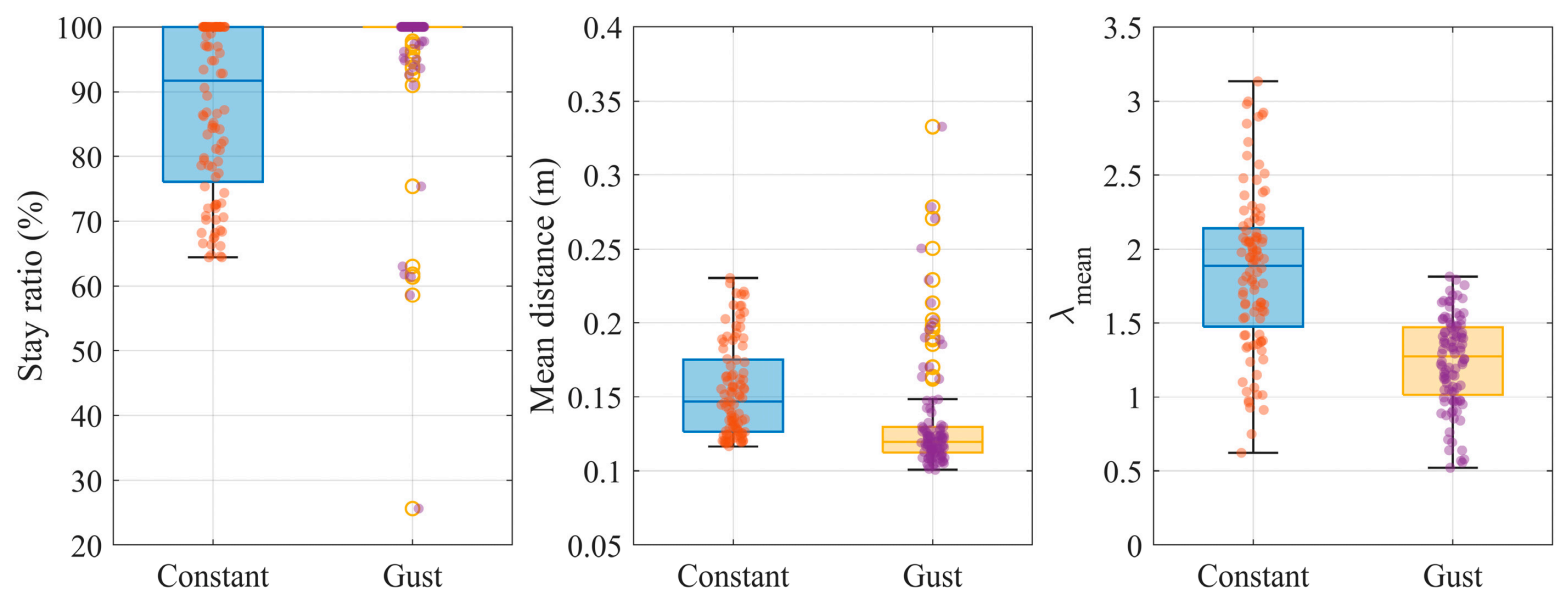
Distributional comparison under constant and gust currents.

Figure 15 gives a distributional comparison of station keeping under constant and gust currents. The boxplots summarize the distributions of the stay ratio, mean target distance, and mean λ over the evaluation episodes, while the overlaid points represent the individual episode results. According to Figure 15, the gust current produces a higher stay-ratio distribution performance. Although a few low-performance results are observed, most gust current episodes remain close to full station keeping. In contrast, the constant current condition gives a lower and wider stay ratio distribution, with a mean value of 87.57 ± 12.81% and a bootstrap 95% CI of [85.06%, 90.02%]. This wider distribution indicates that continuous current disturbances cause higher long-horizon variability than transient gust disturbances. The mean distance distributions further support this observation. Both current conditions yield mean target distance values below the station keeping radius of 0.25 m. Under constant current, the mean distance is 0.155 ± 0.032 m, whereas under gust current it decreases to 0.134 ± 0.041 m. This shows that the robotic fish generally remains close to the target-centered region in both cases, with slightly tighter position regulation under gust current disturbances. The mean λ distribution reveals a clear control-level difference between the two current conditions. Under constant current, the distribution is centered at larger values, with a mean λ of 1.847 ± 0.537. Under gust current, the distribution shifts toward lower values with a mean λ of 1.238 ± 0.308.

It can be shown from these results that the steady disturbances require more sustained directional correction over the episode, while the gust current case can be regulated with a smaller average bias. Therefore, the proposed approach adapts the balance of the control actions according to the temporal structure of the external current disturbance while preserving acceptable station keeping performance in both cases.

### 3.5. Discussion

This study evaluates a TD3–CPG control architecture in three simulated robotic-fish tasks: target reaching, target reaching with obstacle avoidance, and station keeping under external current disturbances. In the evaluations, the proposed TD3–CPG model is compared with DDPG–CPG, PPO–CPG, PID–CPG, and TD3 without CPG under the same target-reaching benchmark. PID–CPG also reaches many targets, but its performance decreases under larger orientation maneuvers. TD3 without a CPG network achieves a relatively high success rate. However, this success is obtained through high-frequency, saturated, and non-sinusoidal joint motions rather than a biomimetic swimming pattern. Therefore, the CPG structure is important for preserving rhythmic and biomimetic control performance.

The main advantage of the proposed framework is that the DRL policy does not directly control the joint angles and motor torques. Instead, it regulates low-dimensional CPG modulation variables, while the sensory-feedback CPG network preserves rhythmic and stable swimming. This hierarchical approach reduces the complexity of the learning problem and provides a more interpretable control structure. The TD3 policy determines the task-dependent modulation, while the CPG network converts these high-level decisions into smooth motions. The target reaching results indicate that the TD3–CPG controller can generalize across different target directions. Front-side targets are reached with compact trajectories, while rear-side targets require larger orientation maneuvers. The TD3–CPG structure handles this process without disrupting the oscillatory locomotion pattern. For obstacle avoidance in target reaching, the same TD3–CPG principle is extended by adding obstacle-related sensory information to the observation vector. The controller achieves high reliability while giving an effective path length. The rectangular obstacle results further show that the proposed controller can handle more structured obstacle configurations. Although some scenarios are more challenging due to narrower gaps and less favorable obstacle placement, the controller generally maintains target-directed motion and safe passing behavior. In the station keeping task, the controller is evaluated under two different current conditions: constant and gust currents. The constant current represents persistent drift under moderate current speeds, while the gust current represents transient rejection and recovery under short-duration and high-intensity pulses. The results show that the controller maintains the robotic fish within the target-centered region for most of the evaluation time under both disturbance types. The constant current case requires stronger and more persistent bias modulation, while the gust current case is generally compensated with shorter correction phases.

Despite these results, the proposed method has some limitations. First, the controller is evaluated using a 2D planar robotic fish model. The model represents surge, sway, and yaw motions and preserves the principal directional, propulsion, and trajectory-level effects required for target reaching, obstacle avoidance, and station keeping. However, it does not represent ascending and descending motions or fully resolve three-dimensional hydrodynamic interactions. Nevertheless, the planar model provides a computationally efficient and control-oriented environment for investigating the interaction between task-dependent observations, reward design, sensory-feedback, TD3 modulation, and CPG-based rhythmic locomotion. In particular, it enables the effects of CPG bias modulation on heading correction, trajectory formation, obstacle clearance, and current-induced drift compensation to be analyzed within a common framework. Transfer to a physical robotic fish may also be influenced by unmodeled hydrodynamic forces, sensor noise, and discrepancies between simulated and real joint dynamics. Therefore, extension of the proposed controller to the full 3D model, followed by experimental validation using a robotic fish prototype, will form the next stage of this research. In addition, the obstacle avoidance results show that failures can still occur when the robot cannot generate a sufficiently early avoidance maneuver. Although the controller produces effective avoidance behavior in most scenarios, unfavorable obstacle configurations may still limit maneuverability. Similarly, the station keeping controller can be further evaluated under stronger and longer-duration disturbances. Overall, the proposed controller provides effective swimming behavior within the investigated operating conditions.

## 4. Conclusions

This study presents a simulation-based investigation of a hierarchical TD3–CPG control framework for a two-joint robotic fish in a 2D planar environment. The framework combines a sensory-feedback CPG network for rhythmic tail motion with a TD3 policy that modulates selected low-dimensional CPG variables according to the task state. The proposed control architecture is evaluated with three tasks: target reaching, obstacle avoidance in target reaching, and station keeping under external current disturbances. A baseline comparison is first conducted for the target-reaching task using TD3-CPG, DDPG-CPG, PPO-CPG, PID-CPG, and only TD3 under the same benchmark. The proposed controller achieves a 100.0% success rate with a Wilson 95% CI of [96.30%, 100.00%]. The TD3-CPG also provides lower arrival time, path stretch, and energy proxy than the baseline models. The designed CPG network produces the necessary rhythmic maneuvers, while the TD3 policy modulates the CPG response according to the task requirements. In task 1, the controller reaches all targets with a 100.0% success rate, and the representative trajectory analysis shows smooth convergence for front side, near rear, and deep rear targets. In task 2, the circular obstacle scenario gives a 98.0% success rate, a 98.0% safe-pass rate, and a path stretch of 1.077 ± 0.041, indicating that obstacle avoidance is achieved with limited geometric overhead. Also, the multiple rectangular obstacle scenarios are evaluated separately and give an overall success rate of 91.7% over 96 trials. In task 3, the controller maintains position regulation under external current disturbances with stay ratios of 87.57 ± 12.81% under constant current and 96.88 ± 10.79% under gust current. The mean target distances remain below the 0.25 m station keeping radius in both cases. These results show that the proposed TD3–CPG policy effectively generates rhythmic modulation for target reaching, obstacle avoidance, and station keeping under different navigation conditions. Future work will focus on extending the proposed framework to more complex obstacle areas and 3D swimming scenarios. Furthermore, an improved observation structure can further enhance coordination between rhythmic motion dynamics. Additionally, complex current conditions with more diverse obstacle geometries and long horizon observations can improve the generalization capability. Finally, a TD3-CPG framework will be evaluated under real-world conditions, and experimental water tank tests will be conducted using a robotic fish prototype to analyze the control framework.

## Figures and Tables

**Table 1:** Comprehensive benchmarking protocol for baseline models.

Target	Direction Range	Num. of Targets	Description
Front side	[−90°, +90°]	50	Forward swimming
Near rear right	[−135°, −90°]	15	Right turning
Near rear left	[+90°, +135°]	15	Left turning
Deep rear right	[−165°, −135°]	10	Right backward swimming
Deep rear left	[+135°, +165°]	10	Left backward swimming
Total	—	100	—

**Table 2:** Controller comparison results for target reaching over 100 evaluation episodes.

Method	Success Rate (%)	Wilson 95% CI	Arrival Time (s)	Path Stretch	Energy Proxy (m^2^/s)
TD3-CPG	100.0	[96.30, 100.00]	14.756 ± 2.633	1.139 ± 0.197	1.325 ± 0.237
DDPG-CPG	94.0	[87.52, 97.22]	15.570 ± 3.860	1.212 ± 0.318	1.479 ± 0.467
PPO-CPG	77.0	[67.85, 84.16]	14.752 ± 3.270	1.140 ± 0.236	1.653 ± 0.649
PID-CPG	89.0	[81.37, 93.75]	14.799 ± 2.159	1.126 ± 0.130	1.480 ± 0.469
TD3	93.0	[86.25, 96.57]	16.441 ± 2.206	1.031 ± 0.036	0.863 ± 0.109

**Table 3:** Target-wise evaluation results of the controllers.

Method	Target	Success	Success Rate (%)	Wilson 95% CI	Arrival Time (s)
TD3-CPG	Front side	50/50	100.0	[92.87, 100.00]	13.087 ± 1.391
	Near rear right	15/15	100.0	[79.61, 100.00]	14.378 ± 1.299
	Near rear left	15/15	100.0	[79.61, 100.00]	15.455 ± 1.651
	Deep rear right	10/10	100.0	[72.25, 100.00]	18.324 ± 2.151
	Deep rear left	10/10	100.0	[72.25, 100.00]	19.056 ± 1.734
DDPG-CPG	Front side	50/50	100.0	[92.87, 100.00]	13.255 ± 1.430
	Near rear right	15/15	100.0	[79.61, 100.00]	15.417 ± 1.840
	Near rear left	15/15	100.0	[79.61, 100.00]	16.593 ± 2.360
	Deep rear right	4/10	40.0	[16.82, 68.73]	23.940 ± 3.258
	Deep rear left	10/10	100.0	[72.25, 100.00]	22.495 ± 3.060
PPO-CPG	Front side	49/50	98.0	[89.51, 99.65]	13.224 ± 1.429
	Near rear right	15/15	100.0	[79.61, 100.00]	15.255 ± 1.373
	Near rear left	9/15	60.0	[35.75, 80.18]	20.050 ± 4.998
	Deep rear right	4/10	40.0	[16.82, 68.73]	19.665 ± 2.643
	Deep rear left	0/10	0.0	[0.00, 27.75]	Failed
PID-CPG	Front side	50/50	100.0	[92.87, 100.00]	13.401 ± 1.418
	Near rear right	13/15	86.7	[62.12, 96.26]	15.599 ± 0.945
	Near rear left	13/15	86.7	[62.12, 96.26]	16.036 ± 1.184
	Deep rear right	6/10	60.0	[31.27, 83.18]	18.293 ± 1.323
	Deep rear left	7/10	70.0	[39.68, 89.22]	18.011 ± 0.795
TD3	Front side	50/50	100.0	[92.87, 100.00]	14.980 ± 1.519
	Near rear right	15/15	100.0	[79.61, 100.00]	17.434 ± 1.400
	Near rear left	15/15	100.0	[79.61, 100.00]	17.896 ± 1.739
	Deep rear right	7/10	70.0	[39.68, 89.22]	19.056 ± 0.785
	Deep rear left	6/10	60.0	[31.27, 83.18]	19.443 ± 1.072

**Table 4:** Paired success and performance differences between TD3-CPG and baseline models.

Method	McNemar *p*-Value	Metric	Mean Difference	Bootstrap 95% CI
DDPG–CPG	0.0412	Arrival time (s)	−1.118	[−1.517, −0.776]
		Path stretch	−0.095	[−0.131, −0.065]
		Path length (m)	−0.523	[−0.709, −0.363]
		Energy proxy (m^2^/s)	−0.102	[−0.139, −0.070]
PPO–CPG	4.49 × 10^−6^	Arrival time (s)	−0.909	[−1.374, −0.534]
		Path stretch	−0.078	[−0.117, −0.047]
		Path length (m)	−0.439	[−0.663, −0.260]
		Energy proxy (m^2^/s)	−0.086	[−0.129, −0.051]
PID–CPG	0.0026	Arrival time (s)	−0.173	[−0.326, 0.000]
		Path stretch	−0.018	[−0.031, −0.004]
		Path length (m)	−0.095	[−0.167, −0.013]
		Energy proxy (m^2^/s)	−0.016	[−0.030, 0.001]
TD3	0.0233	Arrival time (s)	−2.113	[−2.245, −1.973]
		Path stretch	0.086	[0.055, 0.120]
		Path length (m)	0.449	[0.291, 0.623]
		Energy proxy (m^2^/s)	0.427	[0.387, 0.470]

**Table 5:** Randomized initial-state robustness of the TD3–CPG controller.

Metric	Value
Number of trials	50
Initial *x*-position range	[−2.0, 2.0] m
Initial *y*-position range	[−2.0, 2.0] m
Initial heading range	[−180°, 180°]
Target radius	0.15 m
Maximum episode duration	30 s
Successful trials	49/50
Success rate	98.0%
Wilson 95% CI	[89.50%, 99.65%]
Arrival time for successful trials	14.574 ± 3.489 s
95% CI for mean arrival time	[13.572, 15.576] s

**Table 6:** Quantitative performance results of the obstacle avoidance in target reaching over 100 evaluation episodes.

Metric	All Episodes (N = 100)	Wilson 95% CI	Successful Episodes (*n* = 98)	Failed Episodes (*n* = 2)
Success rate (%)	98.0	[93.00, 99.45]	–	–
Collision rate (%)	2.0	[0.55, 7.00]	–	–
Safe-pass rate (%)	98.0	[93.00, 99.45]	–	–
Arrival time (s)	13.884 ± 1.386	–	13.884 ± 1.386	–
Path stretch	1.077 ± 0.041	–	1.077 ± 0.041	–
Minimum clearance (m)	0.551 ± 0.204	–	0.562 ± 0.192	0.048
Average speed (m/s)	0.422 ± 0.009	–	0.422 ± 0.008	0.395 ± 0.014
Energy proxy (m^2^/s)	2.397 ± 0.014	–	2.398 ± 0.013	2.357 ± 0.006
|λ|	1.633 ± 0.459	–	1.635 ± 0.453	1.544 ± 1.002

**Table 7:** Scenario-wise quantitative performance in rectangular obstacle configurations.

Scenario	Geometry (*s*_1_, *s*_2_, gap, *w*, *h*)	Success (%)	Collision (%)	$T_{reach,50}$ (s)	$T_{reach,90}$ (s)	Min. Clearance (m)
1	(0.50, 0.60, 0.70, 0.30, 1.60)	10/12: 83.3%	2/12: 16.7%	14.15	16.09	0.358
2	(0.50, 0.65, 0.70, 0.30, 1.60)	11/12: 91.7%	1/12: 8.3%	14.68	16.49	0.336
3	(0.50, 0.60, 0.90, 0.30, 1.60)	12/12: 100.0%	0/12: 0.0%	15.63	16.36	0.237
4	(0.50, 0.65, 0.90, 0.30, 1.60)	11/12: 91.7%	1/12: 8.3%	14.01	16.96	0.343
5	(0.60, 0.70, 0.70, 0.30, 1.60)	12/12: 100.0%	0/12: 0.0%	15.04	17.26	0.291
6	(0.60, 0.75, 0.70, 0.30, 1.60)	10/12: 83.3%	2/12: 16.7%	14.89	16.98	0.301
7	(0.60, 0.70, 0.90, 0.30, 1.60)	11/12: 91.7%	0/12: 0.0%	14.87	16.69	0.297
8	(0.60, 0.75, 0.90, 0.30, 1.60)	11/12: 91.7%	1/12: 8.3%	15.10	16.54	0.284

**Table 8:** Current disturbance parameters used in station keeping.

Parameter	Constant Current	Gust Current
Initial heading variation	$\psi_{0}\in \left[-10{}^{\circ},+10{}^{\circ}\right]$	$\psi_{0}\in \left[-3{}^{\circ},+3{}^{\circ}\right]$
Current speed magnitude	(0.25, 0.35) m/s	(0.29, 0.31) m/s
Peak current speed	(0.25, 0.35) m/s	$U(1+0.8A_{g})$ m/s
Peak speed range	(0.25, 0.35) m/s	U(1.704−1.896) m/s
Current direction	$\emptyset_{c}\in \left[-15{}^{\circ},+15{}^{\circ}\right]$	$\emptyset_{c}\in \left[-20{}^{\circ},+20{}^{\circ}\right]$
Phase shift	—	$\left[-0.38,\;+0.38\right]$ s
Gust amplitude scale	—	$A_{g}\in \left[0.88,\;1.12\right]$

**Table 9:** Station keeping performance results under different current types.

Current	Stay Ratio (%)	Bootstrap 95% CI	*d_mean_* (m)	*v_mean_* (m/s)	λ*_mean_*	*f_mean_* (Hz)
Constant	87.57 ± 12.81	[85.06, 90.02]	0.155 ± 0.032	0.161 ± 0.031	1.847 ± 0.537	1.389 ± 0.075
Gust	96.88 ± 10.79	[94.61, 98.75]	0.134 ± 0.041	0.142 ± 0.014	1.238 ± 0.308	1.448 ± 0.059

## Data Availability

The original contributions presented in this study are included in the article. Further inquiries can be directed to the corresponding author.

## Appendix A

**Table A1:** Reward function coefficients used in the DRL training process for all task scenarios.

Parameter	Description	Task 1	Task 2	Task 3
$w_{d}$	Distance-progress weight	2.00	2.00	—
$w_{h}$	Heading-alignment weight	1.00	1.30	—
$w_{\nu }$	Target-direction speed weight	0.85	1.00	—
$w_{s}$	Sway penalty weight	0.50	0.50	—
$w_{r}$	Yaw rate penalty weight	0.10	0.10	0.10
$w_{\lambda }$	CPG bias penalty weight	0.015	0.010	—
$w_{∆\lambda }$	CPG bias-rate penalty weight	0.060	0.040	—
$w_{e}$	Tail motion energy penalty weight	0.020	0.020	0.020
$w_{t}$	Per-step time penalty	0.020	0.020	0.010
$R_{g}$	Target-reaching reward	50	—	—
$w_{f}$	Free-space direction reward weight	—	0.80	—
$w_{0}$	Obstacle-approach penalty weight	—	0.40	—
$w_{n}$	Near-obstacle penalty weight	—	1.60	—
$w_{c}$	Clearance recovery weight	—	0.80	—
$R_{cp}$	Safe-clearance and pass reward	—	15	—
$R_{coll}$	Collision penalty with signed value	—	−200	—
$w_{dist}^{SK}$	Station-center distance penalty weight	—	—	4.00
$w_{app}^{SK}$	Station-center approach reward weight	—	—	1.00
$w_{st}^{SK}$	Stay-inside-region reward weight	—	—	2.00
$w_{v}^{SK}$	Station keeping velocity penalty weight	—	—	0.10
